# Forecasting the response to global warming in a heat-sensitive species

**DOI:** 10.1038/s41598-019-39450-5

**Published:** 2019-02-28

**Authors:** Francesca Brivio, Milena Zurmühl, Stefano Grignolio, Jost von Hardenberg, Marco Apollonio, Simone Ciuti

**Affiliations:** 1 Department of Veterinary Medicine, University of Sassari, Sassari, Italy; 2 Institute of Atmospheric Sciences and Climate, National Research Council of Italy, Torino, Italy; 3 Laboratory of Wildlife Ecology and Behaviour, School of Biology and Environmental Science, University College Dublin, Dublin, Ireland

## Abstract

Avoiding hyperthermia entails considerable metabolic costs for endotherms. Such costs increase in warm conditions, when endotherms may trade food intake for cooler areas to avoid heat stress and maximize their energy balance. The need to reduce heat stress may involve the adoption of tactics affecting space use and foraging behaviour, which are important to understand and predict the effects of climate change and inform conservation. We used resource selection models to examine the behavioural response to heat stress in the Alpine ibex (*Capra ibex*), a cold-adapted endotherm particularly prone to overheating. Ibex avoided heat stress by selecting the space based on the maximum daily temperature rather than moving hourly to ‘surf the heat wave’, which minimised movement costs but prevented optimal foraging. By integrating these findings with new climate forecasts, we predict that rising temperatures will force mountain ungulates to move upward and overcrowd thermal refugia with reduced carrying capacity. Our approach helps in identifying priority areas for the conservation of mountain species.

## Introduction

Global temperatures are increasing at unprecedented rates^1^ which may alter selective pressures on animal populations given that thermal balance profoundly affects animal behaviour and population dynamics^2,3^. Temperature can affect the behaviour of endotherms by influencing the quality, distribution and phenology of resources^4,5^. When combined with solar radiation and wind speed, temperature can affect the spatiotemporal variation of the thermal environment experienced by animals (i.e., the operative temperature^6,7^). When the ambient temperature exceeds body temperature, endotherms rely on thermoregulation to avoid lethal heat stress^8^. Thermoregulation accounts indeed for a large portion of the energy spent by endotherms when they cope with warmer conditions^9^. As a result, the trade-offs in energy allocation by endothermic organisms are expected to be driven more by their ability to dissipate heat and avoid hyperthermia than by their ability to harvest energy from the environment, as predicted by the heat dissipation limit theory (HDLT^10^).

Behavioural responses to heat stress can strongly influence spatial distribution of heat-sensitive species facing a warmer climate. Endotherms may buffer themselves against extreme operative temperatures by actively selecting for thermal refuge areas^11^. They can select for woody cover^3,12^ and move to shaded areas^13^, they can prefer open areas on windy days to increase convective cooling^14,15^, or even rest against tree trunks to enhance conductive heat loss^16^. These tactics can reduce thermoregulation costs and increase endotherm ability to allocate energy to other vital processes^3,17,18^. Quantifying the degree to which large herbivores select thermoregulation low-cost areas rather than those where the access to food is higher is a major challenge for ecologists. This trade-off may negatively affect individual fitness as the climate progressively warms. A better understanding of this ecological process should be a priority for species forecasted to be particularly affected by climate change and to define *ad hoc* conservation actions, which include the need to gather fine-scale empirical data, and predict how heat-sensitive species would respond to the new challenges imposed by global warming.

Here, we first combined unique fine-scale data describing spatiotemporal variation in temperature with direct observations of a large cold-adapted endotherm, the Alpine ibex (*Capra ibex*). We then built high-resolution resource selection models to investigate how thermal constraints affect ibex selection of feeding patches during foraging activities. We finally combined our models describing the behaviour of this mountain ungulate with climate change scenarios to predict the response of the species to the increasing temperatures forecasted for the next decades. Alpine ibex is a mountain ungulate that evolved and adapted well to the extreme alpine winter temperatures^19,20^. These adaptations limit the ibex’s ability to dissipate heat and make them prone to overheating. This is particularly the case for males which, weighing twice as much as females, have a lower surface-body mass ratio^21^. Ibex typically cope with high temperatures through behavioural thermoregulation, which entails a reduction of their overall activity and moving to higher elevation, where vegetation quality may be lower^22,23^. A detailed picture of causes and consequences of behavioural responses of mountain ungulates to rising temperatures remains missing and thus limits our ability to understand and predict the consequences of climate change.

We examined resource selection by male ibex in the hours when they are more likely to forage, from early spring to autumn, that is the critical period when they experience the highest environmental temperatures and must acquire energy prior to the rigors of winter^24,25^. We *a priori* formulated the following predictions:

1. Avoidance of higher operative temperatures (i.e., thermal environment experienced by animals^7^) is expected to strongly influence resource selection by ibex, even outweighing food intake in importance. Therefore, when air temperature and solar radiation increase and the thermoregulation becomes critical, ibex are predicted to trade best-food patches for cooler areas where they can minimize radiant heat load.
2. Wind increases convective heat loss, thus improving the animals’ thermal balance in hot conditions^26^. Hence, ibex are predicted to select windy slopes to a greater extent in order to facilitate thermoregulation.
3. Because locomotion is costly and increases metabolic heat production, it should be advantageous to select areas where the overall conditions would be optimal during the hottest hours, rather than moving hourly to ‘surf the heat wave’ and reach cooler areas at upper elevation as the temperature increases during the day. We thus formulate two alternative predictions: (3A) ibex select foraging areas according to maximum daily temperature – that is, they are located where conditions would be optimal when the maximum temperature is recorded; (3B) ibex select foraging areas according to their actual hourly temperature–that is, they are more likely to be located where the conditions are optimal over the short time period (surfing the heat wave).
4. The increasingly warmer summer days forecasted by climate change scenarios may lead ibex to select cooler areas at increasingly higher altitudes. Thus, by combining resource selection models with future climate projections specific of the mountain range occupied by our target species, we predicted a contraction of the extension of ibex suitable area under climate change scenarios. More importantly, the aim of this approach is to characterize and quantify such hotspots that are forecasted to be key for the survival of the species in the region. These hotspots should be targeted by conservation and management actions, and the same approach should be used for the protection of wildlife in mountain ranges, prioritizing those species expected to be particularly hit by global warming.

## Results

### Ibex resource selection: maximum daily temperature is a better predictor than hourly temperature

The large-scale resource selection model including maximum daily temperature as predictor (parameter estimates reported in Table 1) outperformed the alternative model including hourly temperature (Akaike Information Criterion difference: ΔAIC = 273.76). Fixed factors accounted for the entire variability of the response in both models (model with maximum daily temperature: marginal R^2^ = 0.54, conditional R^2^ = 0.54; model with hourly temperature: marginal R^2^ = 0.46; conditional R^2^ = 0.46).

**Table 1:** Generalized linear mixed model parameters estimated for the large-scale resource selection by male ibex observed during 2010–2011 in the Gran Paradiso National Park, Italy.

Variable	β	SE	z	p
max daily temperature	−0.22976	0.03448	−6.67	<0.001
max daily temperature^2^	−0.08209	0.03219	−2.55	0.011
NDVI	−0.14100	0.03334	−4.23	<0.001
NDVI^2^	−0.58023	0.03283	−17.67	<0.001
slope	−0.14091	0.03287	−4.29	<0.001
slope^2^	−0.24852	0.02265	−10.97	<0.001
cos-aspect	−0.00878	0.02073	−0.42	0.672
cos-aspect^2^	−0.06377	0.02836	−2.25	0.025
log-distance from hiking trail	−0.61430	0.02811	−21.86	<0.001
log-distance from hiking trail^2^	−0.08412	0.01073	−7.84	<0.001
distance from safe areas	0.30605	0.03504	8.73	<0.001
distance from safe areas^2^	−0.30209	0.03023	−9.99	<0.001
group size	0.00759	0.03396	0.22	0.823
group size^2^	−0.01940	0.01690	−1.15	0.251
cos-wind direction	0.01592	0.02079	0.77	0.444
cos-wind direction^2^	−0.00246	0.02845	−0.09	0.931
wind speed	0.01377	0.03158	0.44	0.663
wind speed^2^	−0.01248	0.01208	−1.03	0.301
Julian day	−0.16796	0.03138	−5.35	<0.001
Julian day^2^	−0.14056	0.03474	−4.05	<0.001
log-distance from hiking trail × group size	−0.06966	0.01939	−3.59	<0.001
distance from safe areas × group size	−0.28095	0.02690	−10.44	<0.001
max daily temperature × NDVI	−0.68470	0.03983	−17.19	<0.001
cos-aspect × cos-wind direction	0.06186	0.02058	3.01	0.003
cos-aspect × wind speed	−0.06212	0.02196	−2.83	0.005
cos-wind direction × wind speed	−0.02782	0.02029	−1.37	0.170
max daily temperature × Julian day	−0.14895	0.03567	−4.18	<0.001
NDVI × Julian day	−0.54459	0.03526	−15.44	<0.001
slope × Julian day	0.20106	0.02964	6.78	<0.001
cos-aspect × Julian day	−0.06875	0.02137	−3.22	0.001
log-distance from hiking trail × Julian day	−0.08674	0.02075	−4.18	<0.001
distance from safe areas × Julian day	−0.31947	0.03171	−10.07	<0.001
cos-aspect × cos-wind direction × wind speed	−0.06909	0.01974	−3.50	<0.001

**Figure 1:**
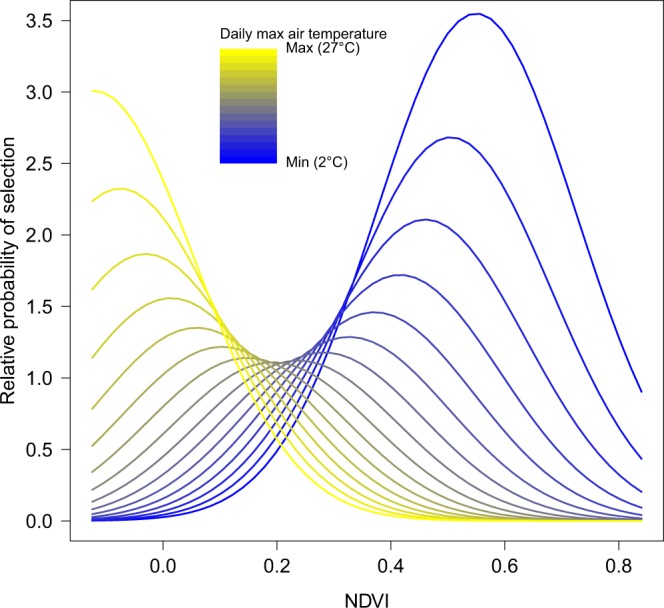
Relative probability of selection for NDVI in interaction with maximum daily temperature as predicted by the large-scale resource selection function, which was built using male ibex observations collected from May to October (2010–2011) in the Gran Paradiso National Park, Italy.

**Figure 2:**
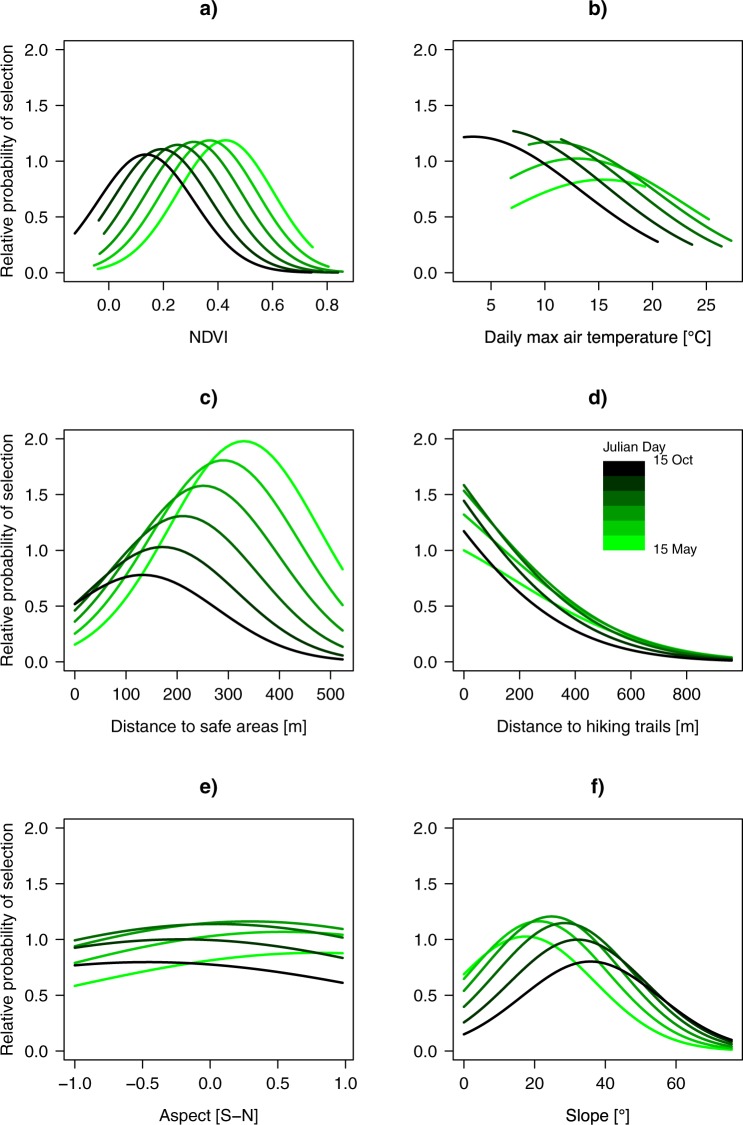
Relative probability of selection as predicted by the large-scale resource selection function, which was built using male ibex observations collected from May to October (2010–2011) in the Gran Paradiso National Park, Italy. Plots depict the effect of the interaction between Julian day and (**a**) NDVI, (**b**) maximum daily temperature, (**c**) distance from safe areas, (**d**) distance from hiking trails, (**e**) aspect (cos-transformed), and (**f**) slope.

**Figure 3:**
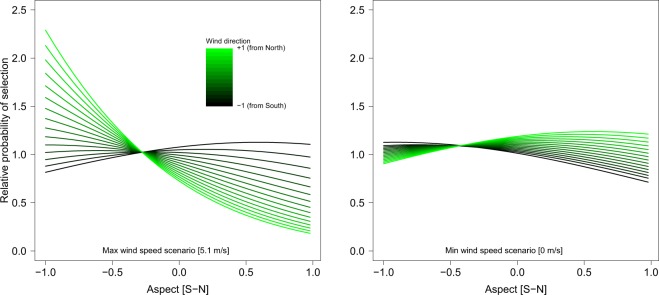
Relative probability of selection for aspect (cos-transformed, x-axes) in interaction with wind direction (different colours represent different scenarios for wind direction) and wind speed (the two plots represent a low and a high wind speed scenario, respectively) as predicted by the large-scale resource selection function, which was built using male ibex observations collected from May to October (2010–2011) in the Gran Paradiso National Park, Italy.

Beta coefficients were extracted and plugged in the exponential resource selection function (RSF) after dropping the intercept, resulting in the resource selection patterns depicted in Figs 1–3 and Fig. S3.1.

Likewise, the small-scale resource selection model including maximum daily temperature as predictor (parameter estimates reported in Supplementary Information 1, Table S1.1) outperformed the alternative model including hourly temperature (ΔAIC = 70.57). Again, fixed factors accounted for the entire variability of the response in both models (model with maximum daily temperature: marginal R^2^ = 0.27, conditional R^2^ = 0.27; model with hourly temperature: marginal R^2^ = 0.26; conditional R^2^ = 0.26).

### Resource selection function (RSF) validation

The 5-fold cross-validation showed that the large-scale resource selection model with maximum daily temperature as predictor had outstanding predictive ability on withheld data (Spearman correlation coefficients: ρ_fold1_ = 0.988, ρ_fold2_ = 0.988, ρ_fold3_ = 0.976, ρ_fold4_ = 0.976, ρ_fold5_ = 0.988; Supplementary Information 2, Fig. S2.1). Compared to the large-scale resource selection model, the small-scale RSF had a weaker - but still acceptable - predictive ability on withheld data (ρ_fold1_ = 0.881, ρ_fold2_ = 0.912, ρ_fold3_ = 0.952, ρ_fold4_ = 0.967, ρ_fold5_ = 0.939; Supplementary Information 2, Fig. S2.2). Thusly, the large-scale resource selection model with maximum daily temperature performed better than any other model as strongly supported by 5-fold cross-validation, and it was eventually used below to predict future ibex resource selection based on projected temperature scenarios.

### RSF predictions

Here we focus on the RSF patterns predicted by the large-scale model by describing 2-way and 3-way interaction terms (parameter estimates in Table 1). The patterns recorded for the small-scale resource selection analysis were similar to those recorded for the large-scale and were reported in full as Supplementary Information (Supplementary Information 1, parameter estimates in Table S1.1).

The interaction between maximum daily temperature and NDVI (Normalized Difference Vegetation Index) was the strongest driver of ibex resource selection, with RSF scores (i.e., relative probability of selection) reaching their highest values (~3.5, Fig. 1; for small scale RSF see Fig. S1.1). Ibex selected areas with higher NDVI whenever maximum daily temperature was lower. When the latter increased, however, ibex moved to areas with lower NDVI values.

Ibex selection of NDVI had a clear temporal pattern (Fig. 2a; for small-scale see Fig. S1.2a); the selection of areas with higher NDVI values occurring in spring was replaced by the selection of areas with lower NDVI values in late summer. Ibex selected warmer sites in spring and cooler ones in summer and early autumn (Fig. 2b). This was not detected by the small-scale analysis, for which the interaction between maximum daily temperature and Julian day was not retained in the final model. Ibex selected areas at greater distance from safe areas in spring, whereas this selection pattern became weaker through summer and early autumn (Fig. 2c; for small scale see Fig. S1.2b).

Male ibex preferred areas closer to hiking trails with variation over time (the interaction distance from hiking trail × Julian day was statistically but not ecologically significant given the effect size: Fig. 2d; see Fig. S1.2c for small-scale). Ibex generally did not select a specific terrain aspect throughout the season (the interaction cos-aspect × Julian day was statistically but not ecologically significant given the effect size: Fig. 2e; see Fig. S1.2d for small-scale) - but see below for the selection of aspect depending on wind conditions. The interaction between slope and Julian day was reported in Fig. 2f (see Fig. S1.2e for small-scale), showing how ibex’s selection of steeper terrain increased at the end of summer.

Large ibex groups were more likely to be located closer to safe areas than solitary individuals and small groups, which tended to stay further away (interaction distance from safe areas × group size: see Fig. S3.1a; and Fig. S1.3a for small-scale), whereas male ibex generally preferred to be closer to hiking trails with the exception of solitary individuals, for which the pattern was weaker (interaction distance from hiking trails × group size: see Fig. S3.1b; and S1.3b for small-scale).

Ibex avoided windy slopes. When winds blowing from the North were stronger, male ibex preferred to stay in south-facing slopes (3-way interaction cos-wind direction × wind speed × cos-aspect Fig. 3; see Fig. S1.4 for small-scale), with a similar (but weaker) trend in opposite conditions (i.e., ibex selecting north-facing slopes when wind was blowing from the South). However, ibex did not select terrain aspect when wind speed was low or absent, regardless of its direction (Fig. 3; see Fig. S1.4 for small-scale). Note that solar radiation was excluded in an early stage of the model selection process (see Methods for full details), resulting into an ibex operative temperature potentially modulated by wind speed but not solar radiation. Ibex did avoid, however, windy slopes, suggesting that air temperature is the main driver affecting ibex spatial choices.

### Predicting ibex resource selection as a response to projected future climate change

**Figure 4:**
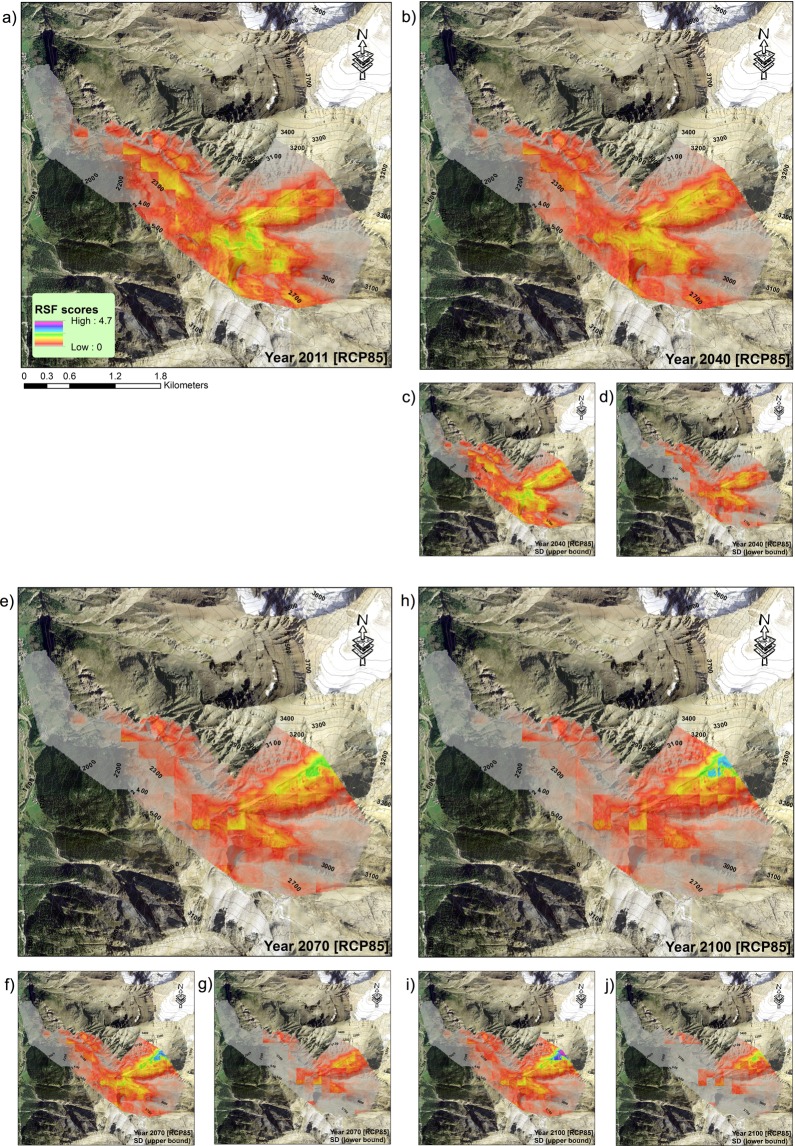
Male ibex resource selection predicted in the Levionaz Valley, Gran Paradiso National Park, Italy, in 2011 (**a**), when this study was carried out, compared to years 2040, 2070, and 2100. Future scenarios are based on temperature projections forecasted by the RCP 8.5 climate models. Large plots depict the average RSF scores across RCP 8.5 simulations (**b**,**e**,**h**), whereas small plots represent upper (**c**,**f**,**i**) and lower standard deviation bounds (**d**,**g**,**j**), respectively. Maps were generated in ArcGIS 10.3 (ESRI 2011). Aerial imagery courtesy Gran Paradiso National Park, Italy.

**Figure 5:**
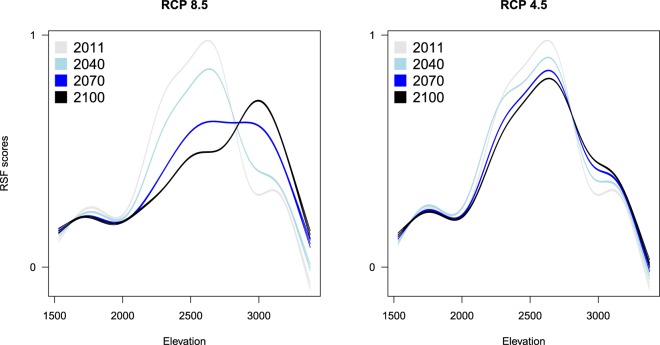
Variation in male ibex resource selection scores plotted against elevation as predicted by RSFs built considering different projected climate change scenarios (RCP 8.5, RCP 4.5). Lines represent predictions by generalized additive linear models (bounded by 95% CI), which were fitted on RSF scores to visualize the shift in ibex selection for higher elevation over time. RSF scores were standardized from 0 to 1 and are proportional to the relative probability of selection.

We predicted summer ibex resource selection for projected climate scenarios (RCP 4.5 and RCP 8.5 climate scenarios from the CMIP5 Climate Model Intercomparison Project) by using the large-scale ibex resource selection model with maximum daily temperature as predictor (Table 1). When considering the worst climate scenario (RCP 8.5), the suitable summer range for ibex is predicted to be strongly contracted (Fig. 4) and located at higher elevations (Fig. 5a). When the less severe climate scenario (RCP 4.5) is considered, although the contraction of suitable habitat is on average less harsh than the one foreseen with RCP 8.5, several RCP 4.5 simulations likewise predict a strong contraction of ibex suitable habitat (Fig. 5b and Supplementary Information 4).

**Figure 6:**
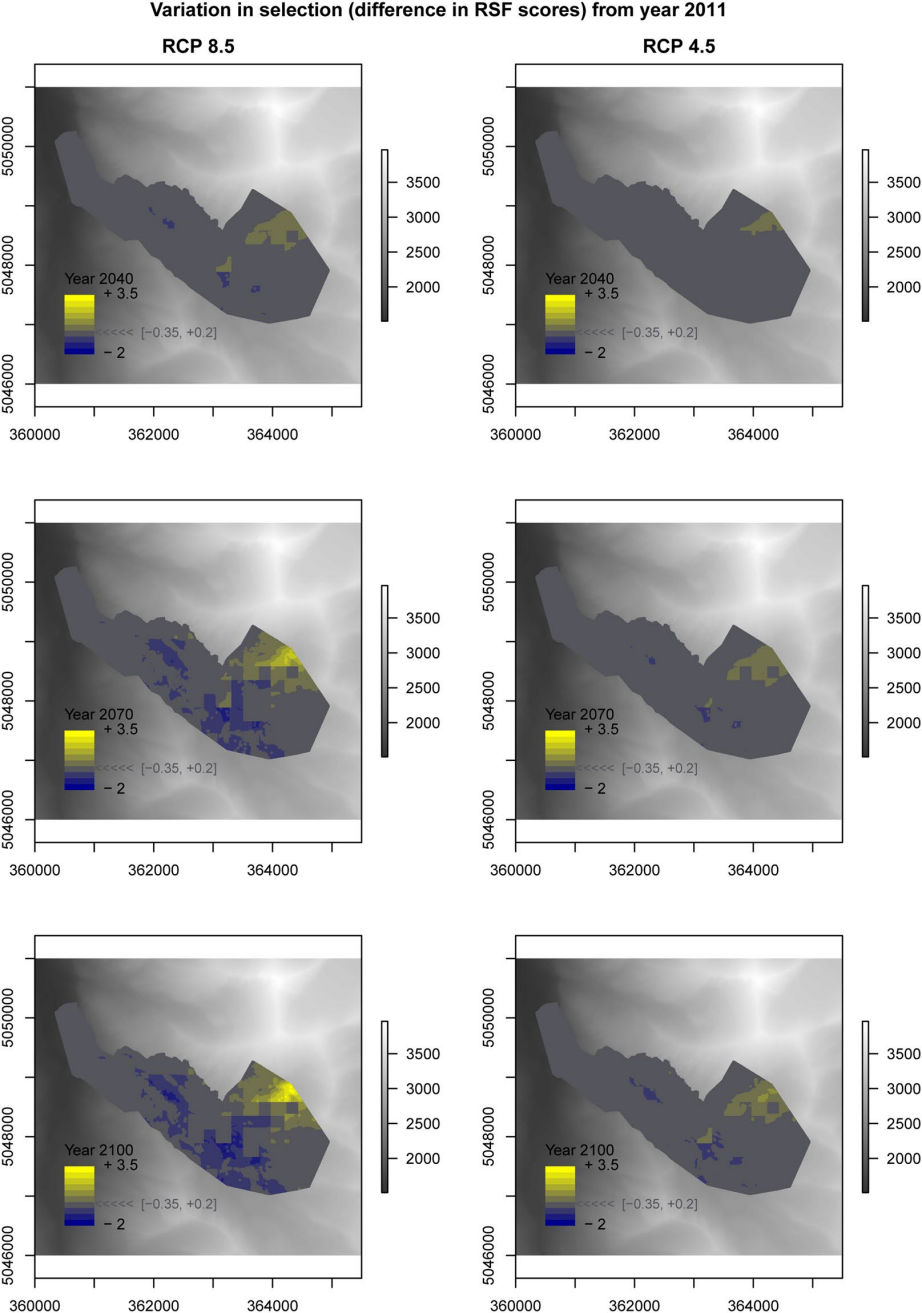
Difference between male ibex RSF scores predicted for years 2040, 2070, 2100 and those recorded for year 2011 (left panels: RCP 8.5 climate change scenario; right panels: RCP 4.5) in the Levionaz Valley, Gran Paradiso National Park, Italy. Positive RSF scores for any given pixels mean that ibex are predicted to select them more than in 2011, whereas negative RSF scores mean that selection is expected to be weaker compared to 2011. RSF scores in grey represent landscape pixels for which we do not expect remarkable change in selection strength by ibex over time (difference in RSF scores ~ = 0). A digital elevation model is shown in the background (legend on the right of each plot, in meters a.s.l.) showing the general elevation gradient of this region, ranging from the valley floor in the West to high mountains in the East and North-East. Maps were generated in ArcGIS 10.3 (ESRI 2011) using a 10 × 10 m Digital Elevation Model (DEM, Regione Valle d’Aosta official data).

By comparing RSF scores projected for 2040, 2070, and 2100 (scenario RCP 8.5) with those predicted for 2011 (Fig. 6), we estimated the fraction of the ibex range that is expected to be more selected during future projections compared to 2011, thus identifying the areas where ibex are expected to concentrate under warmer conditions. The portion of ibex range which is expected to be selected (i.e., where animals are more likely to occur during summer hot days) by males in 2040 corresponds to 38% of the area that could be selected in 2011. This area is predicted to shrink progressively, thus diminishing to 31.0% in 2070 and 26.4% in 2100. The same applies when considering the climate scenario RCP 4.5, even though the phenomenon is expected to be less dramatic (53.2% in 2040, 43.0% in 2070, and 40.8% in 2100).

## Discussion

Our study revealed that resource selection by male ibex under warmer conditions reflected the need to avoid heat stress rather than maximize energy intake, thus corroborating the HDLT (heat dissipation limit theory, *sensu* Speakman & Król^10^). When air temperatures increased, and thermoregulation became critical, ibex traded best food patches for cooler areas (prediction 1). Solar radiation was overtaken in importance by other predictors and excluded by the list of candidate drivers of resource selection in ibex. Contrary to our prediction 2, ibex did not select windy slopes to a greater extent to facilitate heat loss and improve thermal balance. Male ibex spatial behaviour was primarily driven by maximum daily temperatures rather than by those recorded hourly (prediction 3A). This suggests that a heat-sensitive species may cope with warmer temperatures by selecting the space based on where the highest daily temperature is going to be recorded rather than moving hourly to ‘surf (or rather anticipate) the heat wave’. On the one hand, ibex had the chance to move upward by anticipating the heat wave moving up from the bottom of the valley as the day progresses, with the option to move downward in the late afternoon. On the other hand, male ibex had the chance to reduce their overall movement rate and occupy a spot where overheating would be reduced when maximum temperatures are reached. Male ibex adopted the latter strategy. Our models are the first to forecast at unprecedented high-resolution how ibex will respond to global warming – namely by retreating to and concentrating in fewer locations at higher elevation (prediction 4).

### Resource selection during foraging activity

Ibex spent their daily foraging time at the same elevation, which was selected based on the daily maximum temperatures reached there during the central (and hottest) part of the day. In other words, ibex can anticipate hours in advance which locations will have the most favourable temperatures later in the day. As locomotion entails energy expenditure and increases metabolic heat production^17^, animals may achieve a better energy balance by reducing movements and selecting areas where temperatures remain under the critical threshold during the most part of the day, rather than moving continuously to find the best thermal conditions at each moment of the day. How ibex could determine their optimal position in the early morning when the maximum temperature was yet to be experienced is not clear. We argue that ibex can sense temperature trends in advance in the morning by decoding environmental cues. Studies on a variety of taxa, from insects to mammals, showed that animals are able to use environmental cues to predict near-term weather, so as to time their behavioural decisions ahead of impending changes^27–32^. Some species, for instance, can detect approaching storms^33,34^ through barometric pressure. Temperature predictive skills, however, have not been documented in animal species so far. The ability to predict temperature trends - which our study seems to suggest - would be beneficial to species relying on behavioural thermoregulation, because it may help animals to optimize their behavioural decisions (e.g., lowering the costs of locomotion) and to daily select the best pastures where maximum heat stress is tolerable.

**Figure 7:**
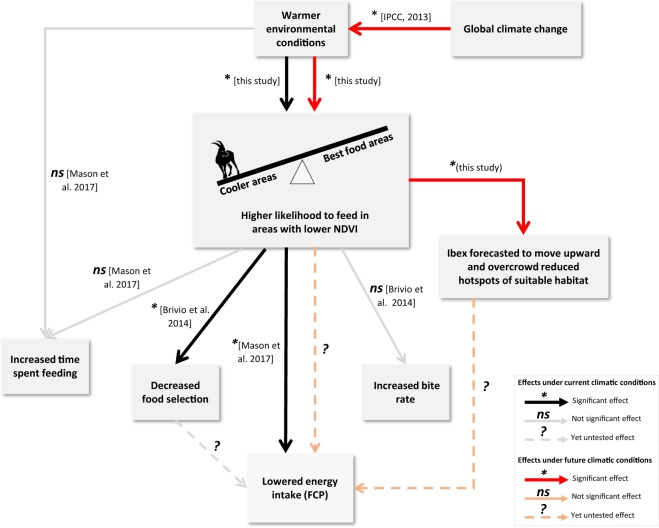
Conceptual diagram of the causality links affecting behavioural decisions and related consequences in a heat-sensitive mountain ungulate such as the Alpine Ibex. NDVI stands for Normalized Difference Vegetation Index, a proxy for vegetation quality and quantity, whereas FCP stands for Faecal Crude Protein, a proxy for animal energy intake. Note that the increase of temperature is considered the main driver here among other potential covariates that are predicted to vary as a consequence of climate change.

The most important driver of resource selection by male ibex was the trade-off between food quality and maximum temperatures. Male ibex selected areas with higher NDVI values whenever maximum temperature was low, and they moved to areas characterised by lower NDVI values when maximum temperature increased. This trade-off was already hypothesised by Mason *et al*.^23^, who analysed time budget data and showed that ibex may respond to variation in seasonal temperatures by migrating towards higher altitudes, where they used areas of lower forage quality. Our spatially-explicit fine-scale analysis clarifies the mechanisms underlying the seasonal movement of male ibex towards higher altitudes and disentangles for the first time the behavioural response of this heat-sensitive species to warmer temperatures. Our study adds significant pieces to the complex puzzle depicting the role of environmental temperature in shaping ibex behavioural patterns and, ultimately, its energy intake (summarised in Fig. 7). We showed that with rising temperatures the outcome of the trade-off between thermoregulation and foraging drove males to feed in areas characterized by lower NDVI. Male ibex cannot compensate for reduced forage quality by increasing bite rate^35^ nor by increasing the amount of time allocated to foraging^23^, whereas it seems that they can only decrease their selectivity in picking food to at least maintain feed intake to certain levels^35^. Consequently, heat stress affects ibex energy intake, precisely during the critical period for their body growth, i.e. spring and summer period (Fig. 7). It is worth noting that we analysed resource selection exactly during the hours when ibex typically (or are strongly expected to) forage, i.e., at dawn and dusk. Ibex did trade thermal cover (e.g., shade within rocky areas with low NDVI values) for optimal foraging exactly when they were supposed to forage early in the morning and late in the evening. Omitting considerations about animal activity in resource selection analysis may lead to misleading results. For instance, if selection of thermal cover occurs only during the resting period (i.e., during the central part of the day), then no direct effects are expected on the energy intake. This is the case of male white-tailed deer (*Odocoileus virginianus*) which selected cooler areas at midday, when they were less active, and areas with greater food quality in the morning and at night, when they typically forage^36^. Consequently, the avoidance of heat stress did not seem to negatively affect the deer’s energy intake. Our results, instead, clearly show that male ibex traded food for thermal cover precisely during the hours when they typically forage, thus arguably compromising food intake.

Ibex did not seem to take advantage of the wind to facilitate their thermoregulation in warmer conditions, but rather avoided windy slopes. Studies on domestic goats^15^ and on other small and large endotherms^26,37,38^ suggested that wind may increase the animals’ ability to dissipate heat by penetrating the pelage and decreasing the air boundary layer surrounding the animal^37^. Contrary to our expectations, however, ibex clearly avoided slopes with stronger wind, particularly if it blew from the North. This suggests that wind was perceived by ibex as a disturbing factor, corroborating the findings on other ungulate species, which were found to avoid windy areas and decrease their activity levels in windy conditions (e.g.,^12,39–42^). Previous research showed that animals significantly increase vigilance^43^ and gregarious behaviour^44^ during windy conditions, as possible responses to an increased perception of predation risk. Strong winds in fact could inhibit the preys’ hearing and olfactory capacity to detect predators^44^. Thus, it seems that in ungulates the negative effects of wind outweigh any potential advantage related to heat dissipation. Such finding pointed out that the potential advantages evolved by ungulate species to reduce predation risk, in this case the avoidance of windy slopes, may be an impediment to face increasingly warmer climate.

### Simulated ibex resource selection as a response to future climate change

Our study offers a rather pessimistic view of the future of mountain heat-sensitive species. Under the more optimistic hypothesis of a stabilisation of the anthropogenic climate forcing during the 21st century (RCP 4.5 scenarios^45^), and arguing that all other covariates will remain unvaried (which is a best case scenario), our simulations predicted that, in 90 years’ time, the extent of the areas more likely selected by ibex during foraging in summer will be less than a half with respect to those selected in 2011. This phenomenon is expected to be more dramatic under the hypothesis of an uninterrupted increase in the evolution of anthropogenic climate forcing (RCP 8.5 scenarios^45^): in 90 years’ time, ibex will more likely be restricted to less than one third of the areas that they selected in 2011. With future rising temperatures the avoidance of heat stress will obviously increasingly influence endotherms’ habitat selection. To avoid the risk of overheating, organisms commonly use thermally buffered microhabitats, such as shaded areas, North-facing slopes and windy areas^3,13–15,46^. Our resource selection analyses, however, suggested that during the foraging activity ibex did not avoid heat stress by selecting either north-facing slopes or windy areas. Additionally, the lack of woody vegetation above 2000 m a.s.l. in the Alps and most other mountain ranges seems to exclude the possibility of finding shelter under the canopy cover. Ibex buffer against high temperatures mainly by moving to higher elevations: our simulations showed that the probability of selection of these areas will increase more and more in 2040, 2070, and 2100. The upslope range shift is a common pattern in the response to global warming for a vast array of animal species, from invertebrate to mammals^47^, and has been documented already in other mountain ungulates^48^. When the species distribution shifts toward the top of mountains, their range-size shrinks, as a result of the progressively diminishing ratio of terrain available and consequent loss of suitable habitat (e.g.^49–51^). In consequence of global warming, then, ibex will forage concentrated in limited areas closer and closer to mountaintops. This will have aftermaths on carrying capacity and on food availability in areas that are already characterised by low vegetation quantity and quality^16^. In turn, the animals’ ability to acquire energy during summer, the critical period for mountain species to acquire energy prior to the rigors of winter, shall undoubtedly be affected, with important repercussions on individual life history and population dynamics (Fig. 7).

### Insights on the improvements for future research aimed at tackling the effect of climate change on endotherm thermoregulation and related spatial tactics

In our study we spatially detailed the thermal environment available to ibex by predicting hourly and daily maximum air temperature at a very fine scale (10 × 10 m). We also combined datasets on spatio-temporal variation in temperatures, solar radiation, wind speed and direction. Estimating the true operative temperature (i.e., thermal environment experienced by animals^7^), however, is the next challenge that has to be faced by future researchers keen to tackle the effect of climate change on endotherm thermoregulation and related spatial tactics. Several sensors used to estimate operative temperature are now accessible to the broader scientific community (initially reviewed by Dzialowski^52^ but now cheaper and more sophisticated technology is available), and such sensors can be deployed across study sites exactly how we did with the temperature sensors but this time by describing the operative temperature experiences by the species under scrutiny. Such operative temperature sensors are able to fully embrace a multivariate problem involving inputs of air temperature, ground temperature, solar radiation, and wind speed and condense them into a single thermal metric on a spatial scale appropriate for the animal^52^. As such, these sensors may take into consideration variations in the solar radiation affected by cloud cover, wavelength, solar elevation, altitude, and atmospheric turbidity^53^, which we were not able to account by means of our analyses.

Our simulations on future ibex distribution did not take into consideration possible changes in the spatial and temporal distribution of other covariates as a consequence of future climate change, except for the air temperature itself. For instance, shift in the distribution and phenology of the vegetation in the Alpine areas^54,55^ could alter the modification in ibex distribution even more than what we predicted with our models. Modifications in plant community structure could also accentuate the heterogeneity of the thermal environment, thus affecting the availability of optimal climatic niches in mountain ungulates^56,57^.

## Conclusions

Our study showed that the impact of global climate change will strongly depend on the actual degree of such change: in the more optimistic scenarios (i.e., anthropogenic climate forcing stabilized), the contraction of ibex suitable habitats will be less severe. To minimise the inevitable negative impact, though, it is imperative to pursue governmental policies aiming to reduce the anthropogenic climate forcing at a global level. Likewise, management strategies minimising disturbance and stress factors need to be implemented at a local level, with the aim to favour the protection of the areas where ibex will find thermal-refugia during hot summers. A slower rate in climate change and reduced stress factors may increase the likelihood for the species’ acclimatisation and adaptation to changing environmental conditions. In this respect, studies on physiological and phenotypic plasticity (e.g., shifting in the activity rhythms) should be promoted to understand how heat-sensitive species are able to cope with the rising temperatures.

## Methods

All data handling and analyses were performed in R 3.3.2^58^, including spatial analyses and visualization, whereas some of the maps depicting the predicted response of ibex to global warming were done in ArcGIS 10.3 (ESRI 2011).

This study complied with all national and regional laws dealing with ethics and animal welfare. Ibex capture and manipulation protocol was approved by the Italian Ministry of Environment (Protoc. No. 25114/04).

### Study area and ibex population

Ibex direct observations were carried out in the Levionaz Valley (~1700 ha, 1700–3300 m a.s.l.), a steep glacial valley of the Gran Paradiso National Park (GPNP: 45°35′N, 7°12′E), north-western Italy. The valley bottom was covered by meadows (mainly *Festuca* and *Poa* spp.) and patches of conifers (mainly larch, *Larix decidua*). The area located above the tree line (2300 m a.s.l.), that is the area used by the ibex population, was dominated by rocks, scree slopes, meadows and grassland^59,60^.

Ibex randomly selected for the long-term longitudinal study running in the GPNP since the 1990s were chemically immobilized and marked (colour-coded ear tags and/or collars) by park rangers assisted by a veterinarian^61^ in order to be individually recognisable during field observations. Age of animals was estimated through horn annuli counting^62^. We focused on the ecology of 57 individually-recognizable male ibex monitored in 2010 and 2011, when park rangers estimated the autumn population size to 133 (51 males, out of which 44 were marked) and 151 individuals (60 males, out of which 52 were marked), respectively. We thus directly observed and collected spatial data on >85% of the male population present in the region at the time of the study. The only other widespread large herbivore in the study area was chamois (*Rubicapra rupicapra*). The occasional presence of a wolf pack (*Canis lupus*) was recorded in the National Park during the study period, although ibex was a secondary prey item for this large predator (the consumption of ibex carcasses constituting only 8%-14% of wolf diet^63^). Predation by golden eagles (*Aquila chrysaetos*) was limited to ibex kids. Hunting was not permitted in the National Park.

### Ibex location data

Ibex location data were obtained by means of direct observations of individually-recognizable male ibex carried out from early May to late October during two consecutive years (2010–2011). Individually-recognizable male ibex were systematically observed and located during field surveys aimed at contacting all the individuals of this population. More in detail, we walked 10 hiking trails at dawn (mostly between 5 and 9 am) and dusk (mostly between 4 and 8 pm) for a total of 163 days in the field (n = 68 days in 2010, n = 95 days in 2011). Observations were primarily concentrated during daily peaks of activity corresponding to dawn and dusk due to our need to locate ibex when they engage on foraging activities^22^. We provided full details on the spatio-temporal resolution of the ibex observations sighted from the 10 hiking trails in the Supplementary Information 5.

Observed ibex groups were defined as one or more animals with inter-individual distance lower than 50 meters. For each group observed, we recorded time and date of observation, group size, and identity of marked individuals by reading the colour-coded ear-tags. Group spatial coordinates (i.e., the geometrical centre of each group) were calculated through the combination of a GPS handheld unit (Garming CSx60), a compass (Eyeskey Optics) and a rangefinder (Leica 7 × 42). Double group counts were not possible within the same day due to the overwhelming presence of individually-recognizable individuals. We eventually collected 3,275 observations of 57 marked male ibex sighted within 1,122 groups of males. Full details on ibex observations can be found in the Supplementary Information 5.

### Environmental covariates expected to drive ibex resource selection

Air temperature (°C), solar radiation (W/m²), wind speed (m/s) and wind direction (cosine-transformed to range between -1 with wind blowing from the South and +1 with wind blowing from the North) were recorded every hour by the closest weather station (Pont Station, 45°31′36.62N, 7°12′03.36 E; 1951 m a.s.l.; Regione Valle d’Aosta, official data).

Fine-scale temperatures were recorded hourly in the Levionaz Valley using temperature loggers (iButton DS1922L, Maxim Integrated, n = 15 in 2010, n = 17 in 2011) stratified by elevation and hydro-geographic sectors corresponding to different micro-climatic conditions (see Supplementary Information 6, Fig. S6.1 and S6.2). Data from temperature loggers were combined with those collected by the weather station and used to build interpolation models predicting hourly and maximum daily temperature for each of the 10 × 10 m pixels in the study area at any given day within the study period (see Supplementary Information 6 for full details).

To estimate vegetation quality and quantity we used the Normalized Difference Vegetation Index (NDVI), which has been widely used to depict forage productivity in mountain ungulates^24,51,64–66^, and proved to strongly correlate with faecal crude proteins in ibex^23^. NDVI was acquired by the Moderate-resolution Imaging Spectroradiometer (MODIS) on board of the AQUA satellite (16-day-composites from daily data recorded at a 250 × 250 m pixel size).

A 10 × 10 m Digital Elevation Model (DEM, Regione Valle d’Aosta official data) was used to generate same-resolution raster files for terrain aspect (cosine-transformed to range from −1 to 1), terrain slope (in degrees), and terrain ruggedness (in meters, calculated *sensu* Riley *et al*.^67^).

A 4-level categorical land cover map based on aerial image interpretation and validated by ground surveys was provided by the GPNP (GPNP, official data). Levels were defined as follows: meadows and grassland, woods and bushes (i.e., larch and Swiss stone pinewoods, pioneer woods, invasive bush, bushes), screes and rocks, and other (i.e., abandoned crop fields, urban areas/infrastructure).

Based on previous studies on the anti-predator behaviour of mountain ungulates^68–70^, we defined safe areas as rock and scree sites with a slope steeper than 45°. We thus calculated a raster with the distance from >45° steep safe areas as a proxy for predation risk. We repeated the same procedure with a different threshold (distance from areas with >30° slope) because, to the best of our knowledge of ibex ecology, our definition of safe areas slightly differed from the one currently accepted. Finally, we created a raster of the distance from the closest hiking trail as a proxy for human disturbance, along with the estimate of the average number of hikers using that trail (GPNP, official data). Hiking trails have been historically outlined to avoid rugged terrains, to maximize wildlife sighting, and typically lay at bottom of U-shaped valleys. Therefore, hikers walking on the trails are easily detected by ibex present in the valley, from here the clear association of trails to human presence by ibex^71^.

### Setting up the scene for resource selection analyses

We modelled resource selection by male ibex using a presence/available design^72^, with resources sampled both where recognizable ibex were located (presence data, hereinafter referred to as ‘used’ locations) and at randomly selected locations (hereinafter referred to as ‘available’ locations) representing the resources available to ibex^73–75^.

The extent of the area in which we sampled random available locations defined the scale of the analysis (*sensu* Boyce^76^ and Thurfjell *et al*.^74^). To provide a satisfactory overview of ibex resource selection, analyses were carried out on two spatial scales^73,77^. Large-scale availability was sampled within the population-level home range calculated with ibex locations (minimum convex polygon, MCP, 100%; Supplementary Information 5, Fig. S5.2). The small-scale availability was sampled at the individual level by generating a circular buffer around each ibex location, the size of the buffer being a function of monthly ibex mobility derived from satellite telemetry data (see Supplementary Information 7 for full details). The small-scale analysis, therefore, defines a set of available resources that are reachable by each monitored ibex in a given month, whereas the large-scale analysis is less restrictive and considers the resources available to each monitored ibex throughout the whole population range (*sensu* Thurfjell *et al*.^74^).

When depicting random availability in resource selection analyses, the number of available random points per used point may affect parameter estimates, which can be particularly unstable when sample size for random availability is low^74,75^ (see also Box 2 in Roberts *et al*.^78^). We thus ran a sensitivity analysis for each of the two spatial scales to define the minimum number of random available points that needed to be associated to each used location. Results of the sensitivity analysis suggested the use of 15 random points per used location in the large-scale analysis, and 13 random points per used location in the small-scale analysis (see Supplementary Information 8 for full details).

Available locations (0 s) were thus paired to used ones (1 s) and each pairing (ratio 1:15 and 1:13 for large- and small-scale, respectively) was assigned a unique identification code (stratum-ID). The individual attributes of each ibex observed, i.e. male identity, age, group identity, group size, and date and time of observation, were assigned to its respective used location, as well as to its corresponding available locations. We extracted environmental covariates based on spatial location for both used and available locations, and assigned NDVI and air temperature data based on spatial coordinates, date and time of observation.

### Ibex resource selection functions: full model writing and simplification

We built resource selection functions (RSFs) which were assumed to take an exponential form^72^. RSF coefficients were estimated by generalized linear mixed models (GLMMs) with binary response variable (used = 1, available = 0). GLMMs were fitted using the *glmer* function of the *lme4* package^79^. The stratum-ID (identifying each used location paired with its random available locations) was nested within the individual-ID (identifying the individual ibex) and was included as a random intercept in the model, whereas the group-ID (identifying the group where the marked ibex was observed) was included as a (crossed) random intercept. All numerical predictors were scaled [(x - mean)/SD] prior to running any model.

As introduced earlier, we modelled ibex resource selection at two different scales (population level, i.e. large-scale; individual level, i.e. small-scale). In both cases, we *a priori* created a full model structure including two- and three-way interactions based on our understanding of ibex ecology and the main expectations as to the effect of predictors in driving ibex habitat selection. The predictor variables included in the full model structure were reported in the Supplementary Information 9, Table S9.1. Given that these *a priori* defined model structures could potentially include collinear predictors and that the number of parameters could possibly exceed that which could be estimated with our sample size (i.e., failing to convergence), we applied a step-by-step protocol to remove collinear predictors and reasonably simplify the model structure.

Predictors were screened for collinearity (Pearson correlation matrix) and multicollinearity (Variance Inflation Factor^80^, with thresholds set to |r_p_| = 0.7 and VIF = 3, respectively). As elevation was collinear with NDVI, we retained the latter because of its relevance in ibex ecology^65^. Maximum daily temperatures and hourly temperatures were also collinear. However, to test the hypothesis that ibex may have evolved the ability to select habitats according to daily temperature trends, we built alternative models using either the hourly temperature or the maximum daily temperature as a covariate, for both spatial scales. As several other pairs of covariates were found to be collinear, they were screened with a machine learning method (*randomForest* package, n = 500 decision trees^81^) to select candidate predictors for the four final models (i.e., two spatial scales associated with two alternative temperature metrics). Consequently, distance from safe areas defined by a slope steeper than 45° outranked the distance defined by a slope steeper than 30°; likewise, slope outranked the terrain ruggedness index. Julian day was found to be the best predictor of temporal variation in resource selection and we dropped the other time predictors (e.g., month of the year, season). Finally, while in large-scale models the (continuous) time of the day was preferred over its categorical version (dawn, day, dusk), it was dropped in small-scale models because it was collinear with hourly air temperatures.

Once the collinear predictors were removed, we further simplified the structure of our *a priori* starting models by running a second random forest analysis with all the remaining candidate predictors. To this end, the following predictors were also dropped: ibex age, the estimated number of hikers along hiking trails, time of the day and land cover. Finally, a manual step AIC procedure was run to remove the predictors that contributed to the increase of model AIC (i.e., worst model performances). With this procedure we removed: (i) solar radiation in all alternative models; (ii) the interaction between hourly temperature and Julian day in the large-scale model with hourly temperature; (iii) the interaction between air temperature (in both metrics) and Julian day in small-scale models.

We finally fit and compared the obtained simplified versions of our alternative *a priori* models built with different air temperature metrics using AIC. R-squared has a limited relevance in presence/available designs, because the model’s ability to explain the variability of the response is affected by the number of available locations, which means that R^2^ is underestimated. As we aimed to correctly classify presence (and not available) data^73^, we calculated R^2^ for our mixed models (*rsquaredGLMM* function of the *MuMIn* package) in order to compare the performances of the two alternative models (i.e., the higher the R^2^, the higher the relative - but not absolute - variability explained) and to provide a clue as to the variability explained by fixed and random effect in our models (see below for more details on model validations). For mixed-effect models, R² can be categorized into two types: marginal R_GLMM², which represents the variance explained by fixed factors, and conditional R_GLMM², which represents the variance explained by both fixed and random factors (i.e. the entire model^82^).

### Final step of resource selection analyses: RSF validation and visualization of predictions

RSF models estimated with presence/available data create unique problems for evaluating model predictions, because presence/available data are truly not as binary as presence/absence data^73^. RSFs were thus validated using the 5-fold cross-validation method developed for presence/available designs by Boyce *et al*.^73^, which involved calculating the correlation between RSF ranks and area-adjusted frequencies for a withheld sub-sample of data, that is 1/5 of the data in a 5-fold cross-validation scheme. We investigated the pattern of predicted RSF scores for partitioned testing data (presence-only) against categories of RSF scores (10 bins). A Spearman rank correlation between area-adjusted frequency of cross-validation points within individual bins and the bin rank was calculated for each cross-validated model. A model with good predictive performance would be expected to be one with a strong positive correlation, as more used locations (area-adjusted) would progressively fall into higher RSF bins^73,83^.

Beta coefficients estimated by the top ranked GLMMs (large-scale, small-scale) were eventually plugged in the resource selection function to obtain RSF scores, which are proportional to the probability of selection. We assumed the RSF to take the form *w(x)* = *exp(β*_1_ × *x*_1_ + *β*_2_ × *x*_2_ + *…* +*β*_*n*_ × *x*_*n*_), where *β*_1_ to *β*_*n*_ are coefficients estimated by GLMM, which are associated with a vector x of environmental variables *x*_1_ to *x*_*n*_, respectively^72,84^.

### Predicting ibex resource selection as a response to predicted future climate change

To predict ibex habitat selection in response to future climate change, we used our best large-scale ibex resource selection model, which had maximum daily temperature as a predictor. Full details on the model equation are provided in the result section, where the structure of the best model was identified. We compared predictions of our models between 2011 (when data were collected) to representative future years 2040, 2070, and 2100. We applied the equation of our RSF model to predict the relative probability of selection depending on observed (2011) and projected (2040, 2070, 2100) air temperatures.

We used projections of future maximum daily temperature produced with global climate models participating in CMIP5 (Coupled Model Intercomparison Project phase 5). For each model output, at its native resolution, we selected the monthly average maximum daily temperatures in the month of August, at the grid point closest to our study site (45.58 latitude, 7.23 longitude). We considered the two future “representative concentration pathways” (scenarios) RCP 4.5 (n = 38 models) and RCP 8.5 (n = 30 models^45^). The first scenario envisioned the stabilization of climate forcing during the 21st century, whereas the second is more extreme and corresponds to a “business as usual” scenario of the future evolution of anthropogenic climate forcing, with an uninterrupted increase. It is worth noting that we included neither the more moderate RCP 2.6 “mitigation” scenario (which would require the application of drastic mitigation measures), nor the intermediate scenario RCP 6.0 available for CMIP5. RCP 4.5 and RCP 8.5 were chosen in that they represented a likely and a very extreme scenario, respectively. All data were downloaded from the ESGF (Earth Science Grid Federation) archive data nodes (http://esgf.llnl.gov). Resolutions of and reference information on the models used are reported in Supplementary Information 10, Table S10.1. Further details on their configuration and features can be found in the PCMDI data website (http://www-pcmdi.llnl.gov/) and in Chapter 9 of the latest International Panel on Climate Change (IPCC) Assessment Report^1^. Due to the high uncertainty of the projections of future temperatures, particularly at a regional scale^1,85^, the use of a large ensemble of global climate models considering different scenarios of anthropogenic forcing is crucial to gauge future uncertainty.

When predicting RSF scores over space and time, we needed to feed the equation with pre-conditions. To create the scenario for 2011, for instance, we selected the Julian day 233 (21st August 2011), i.e. the hottest day recorded in the study site over that period. Wind direction and speed were set to median values for the month of August 2011, while group size was set to 2 individuals, following the same rationale (median group size for that period of the year). Our aim was to describe the variation in the availability of suitable habitats for ibex when temperatures reach their peak, i.e., when ibex must cope with thermoregulation and habitat selection in challenging situations that are expected to be increasingly common in the future.

For each model simulation we calculated the difference between the temperature predicted for the month of August of the years 2040, 2070 and 2100 with respect to that recorded in August 2011, obtaining ΔT_2040_ (mean ± SD, in Celsius degrees: 0.20 ± 3.05 for RCP 4.5, 2.11 ± 2.22 for RCP 8.5), ΔT_2070_ (mean ± SD: 1.65 ± 3.20 for RCP 4.5, 6.03 ± 3.45 for RCP 8.5), and ΔT_2100_ (mean ± SD: 2.73 ± 2.94 for RCP 4.5, 8.39 ± 3.51 for RCP 8.5), respectively. We computed the future air temperature for our study area by adding the ΔT_2040_, ΔT_2070_, and ΔT_2100_ values to the temperatures derived from the interpolation model. By computing these differences based on specific years instead of using the difference between averages over longer time frames, we implicitly added a representation of model inter-annual variability to the ensemble spread. We eventually obtained RSF scores predicted for the years 2011, 2040, 2070, 2100, obtaining for any given future year 38 and 30 RSF predictions for the RCP 4.5 and RCP 8.5 scenarios, respectively. We finally created maps depicting average and standard deviation of RSF projections depending on the different simulations.

## Supplementary information

Supplementary Information

## Data Availability

The datasets generated and analysed during the current study are available in the Dryad Digital Repository, 10.5061/dryad.585b5k5.
