# Supplementary material for: Forecasting the response to global warming in a heat-sensitive species
Source: Sci Rep. 2019 Feb 28;9:3048. doi: 10.1038/s41598-019-39450-5 (PMC6395821; doi:10.1038/s41598-019-39450-5)
Supplement: Supplementary file 1 — Supplementary Information [file 41598_2019_39450_MOESM1_ESM.pdf]

Forecasting the response to global warming in a heat-sensitive species

Francesca Brivio[1*], Milena Zurmühl[3], Stefano Grignolio[1], Jost von Hardenberg[2], Marco Apollonio[1], Simone Ciuti[3]

[1] Department of Veterinary Medicine, University of Sassari, Sassari, Italy.

[2] Institute of Atmospheric Sciences and Climate, National Research Council of Italy, Torino, Italy.

[3] Laboratory of Wildlife Ecology and Behaviour, School of Biology and Environmental Science, University College Dublin, Dublin, Ireland

Supplementary information

1 - Small scale resource selection analysis.. 3

2 – Resource Selection Function k-fold validation 8

3 - Large scale resource selection analysis: additional results 10

4 – Resource Selection Function projections for RCP 4.5... 11

5 – Additional maps and plots on data collection.. 12

6 - Temperature interpolation models.. 17

7 - Calculation of buffer sizes needed to depict random availability in small-scale resource selection analysis. 22

8 - Sensitivity analysis aimed at defining the minimum number of random available points to be associated with ibex presence data in resource selection analyses...... 24

9 - Environmental covariates used in the ibex resource selection analyses. 27

10 - Additional information on climate models. 28

1 - Small scale resource selection analysis

Table S1.1 - GLMM beta estimates for small-scale resource selection by male ibex observed from 2010 to 2011 in the Gran Paradiso National Park, Italy. Beta coefficients were plugged in the exponential resource selection function RSF after dropping the intercept, resulting in the resource selection patterns depicted in Figs. S1.1-4.

Variable	β	SE	z	p
daily max temperature	-0.07441	0.02632	-2.83	0.005
daily max temperature^2	0.03951	0.01798	2.20	0.028
NDVI	0.10311	0.02686	3.84	< 0.001
NDVI^2	-0.18128	0.01842	-9.84	< 0.001
slope	-0.09189	0.03255	-2.82	0.005
slope^2	-0.21831	0.02273	-9.60	< 0.001
cos-aspect	-0.04103	0.01991	-2.06	0.039
cos-aspect^2	-0.08840	0.02731	-3.24	0.001
log-distance to hiking trail	-0.66192	0.03023	-21.89	< 0.001
log-distance to hiking trail^2	-0.10188	0.01112	-9.16	< 0.001
distance to safe areas	0.30727	0.03588	8.56	< 0.001
distance to safe areas^2	-0.24174	0.02575	-9.39	< 0.001
group size	0.11259	0.03429	3.28	0.001
group size^2	-0.04395	0.01676	-2.62	0.009
cos-wind direction	-0.00963	0.02001	-0.48	0.630
cos-wind direction^2	0.00868	0.02715	0.32	0.749
wind speed	0.07274	0.03065	2.37	0.018
wind speed^2	-0.03178	0.01169	-2.72	0.007
Julian day	0.07186	0.02617	2.75	0.006
Julian day^2	-0.01173	0.02881	-0.41	0.684
log-distance to hiking trail × group size	-0.07108	0.01828	-3.89	< 0.001
distance to safe areas × group size	-0.23745	0.02395	-9.91	< 0.001
daily max temperature × NDVI	-0.20654	0.02367	-8.73	< 0.001
cos-aspect × cos-wind direction	0.04682	0.01984	2.36	0.018
cos-aspect × wind speed	-0.05592	0.02104	-2.66	0.008
cos-wind direction × wind speed	-0.01615	0.01955	-0.83	0.409
NDVI × Julian day	-0.18990	0.02511	-7.56	< 0.001
slope × Julian day	0.13668	0.02778	4.92	< 0.001
cos-aspect × Julian day	-0.06687	0.01965	-3.40	< 0.001
log-distance to hiking trail × Julian day	-0.06134	0.01936	-3.17	0.002
distance to safe areas × Julian day	-0.26940	0.02714	-9.93	< 0.001
cos-aspect × cos-wind direction × wind speed	-0.05626	0.01892	-2.97	0.003

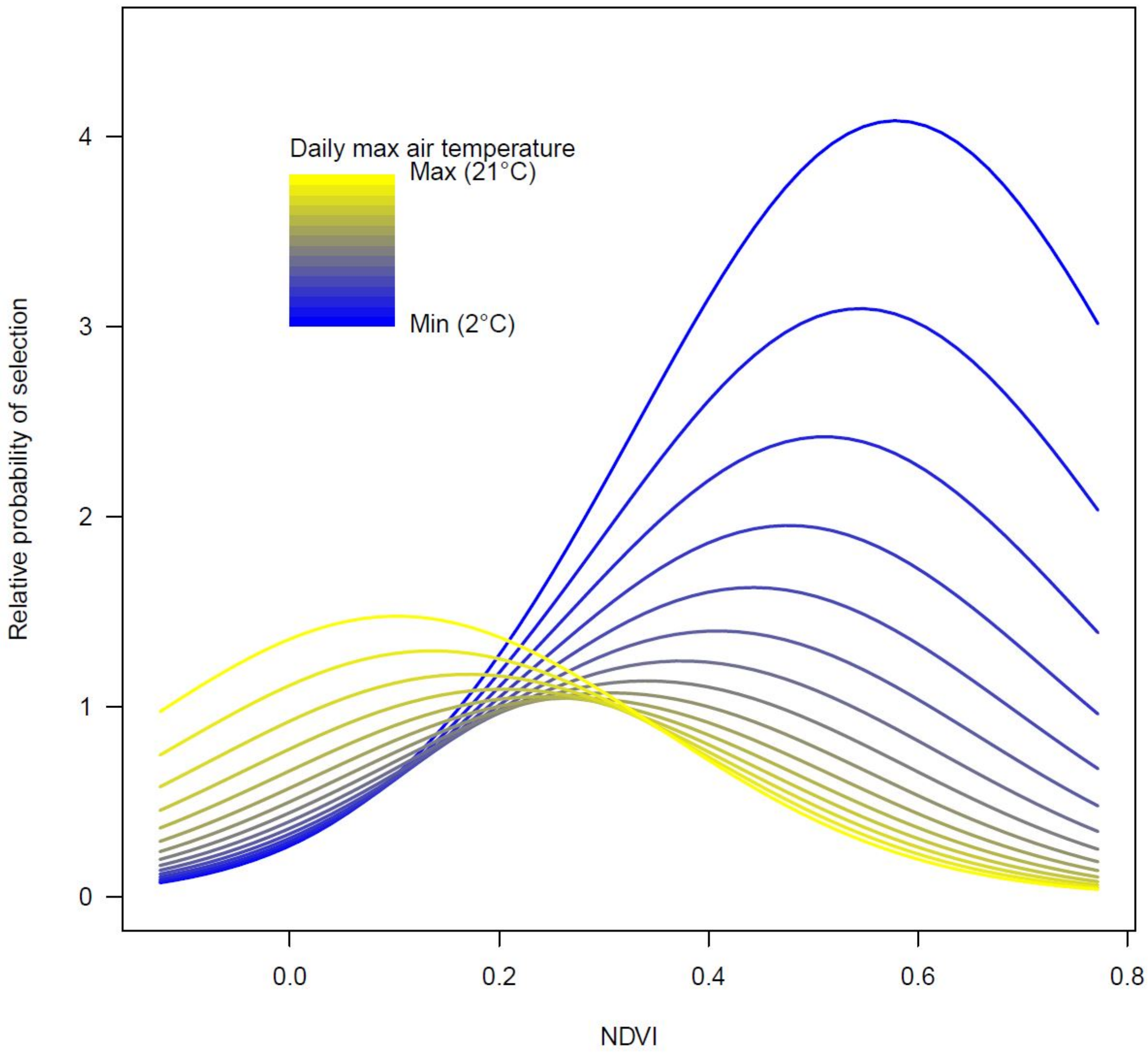

Figure S1.1 - Relative probability of selection for NDVI interacted with daily max air temperature as predicted by the small-scale resource selection function, which was built using male ibex observations collected from May to October (2010-2011) in the Gran Paradiso National Park, Italy.

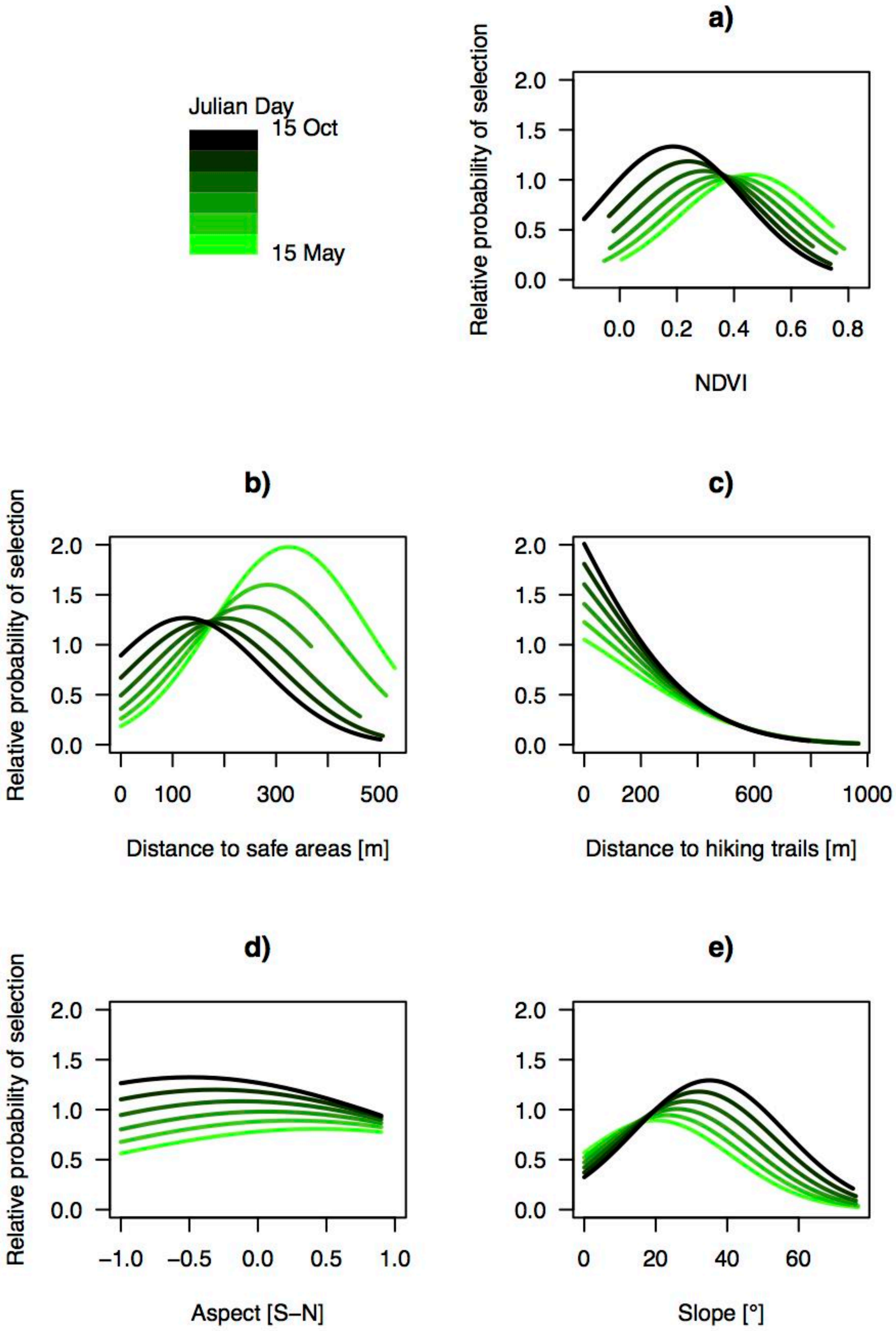

Figure S1.2 - Relative probability of selection as predicted by the small-scale resource selection function, which was built using male ibex observations collected from May to October (2010-2011) in the Gran Paradiso National Park, Italy. Plots depict the effect of the interaction between Julian day with a) NDVI, b) distance to safe areas, c) distance to hiking trails, d) aspect (cos-transformed), and e) slope.

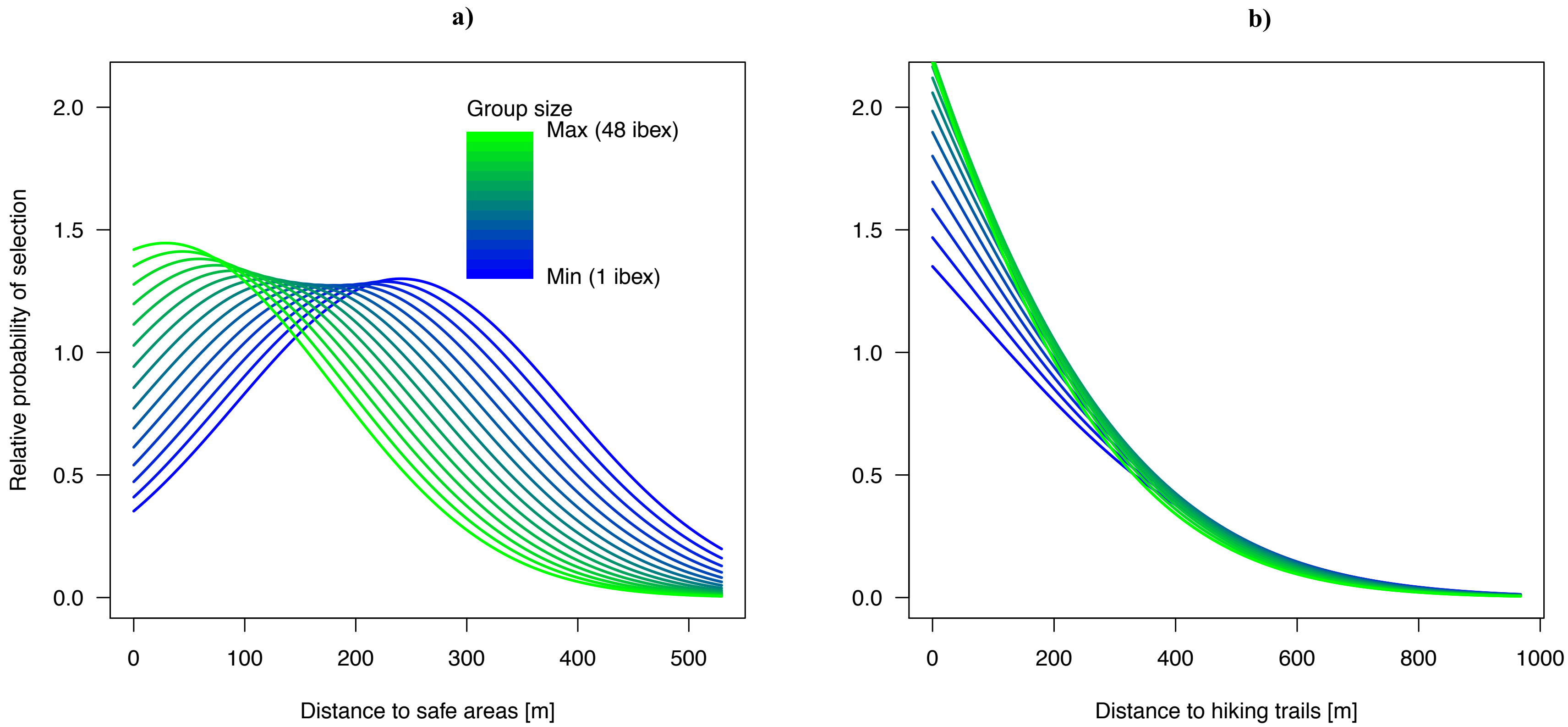

Figure S1.3 - Relative probability of selection for the distance to safe areas (a) and the distance to hiking trails (b), both interacted with ibex group size, as predicted by the small-scale resource selection function, which was built using male ibex observations collected from May to October (2010-2011) in the Gran Paradiso National Park, Italy

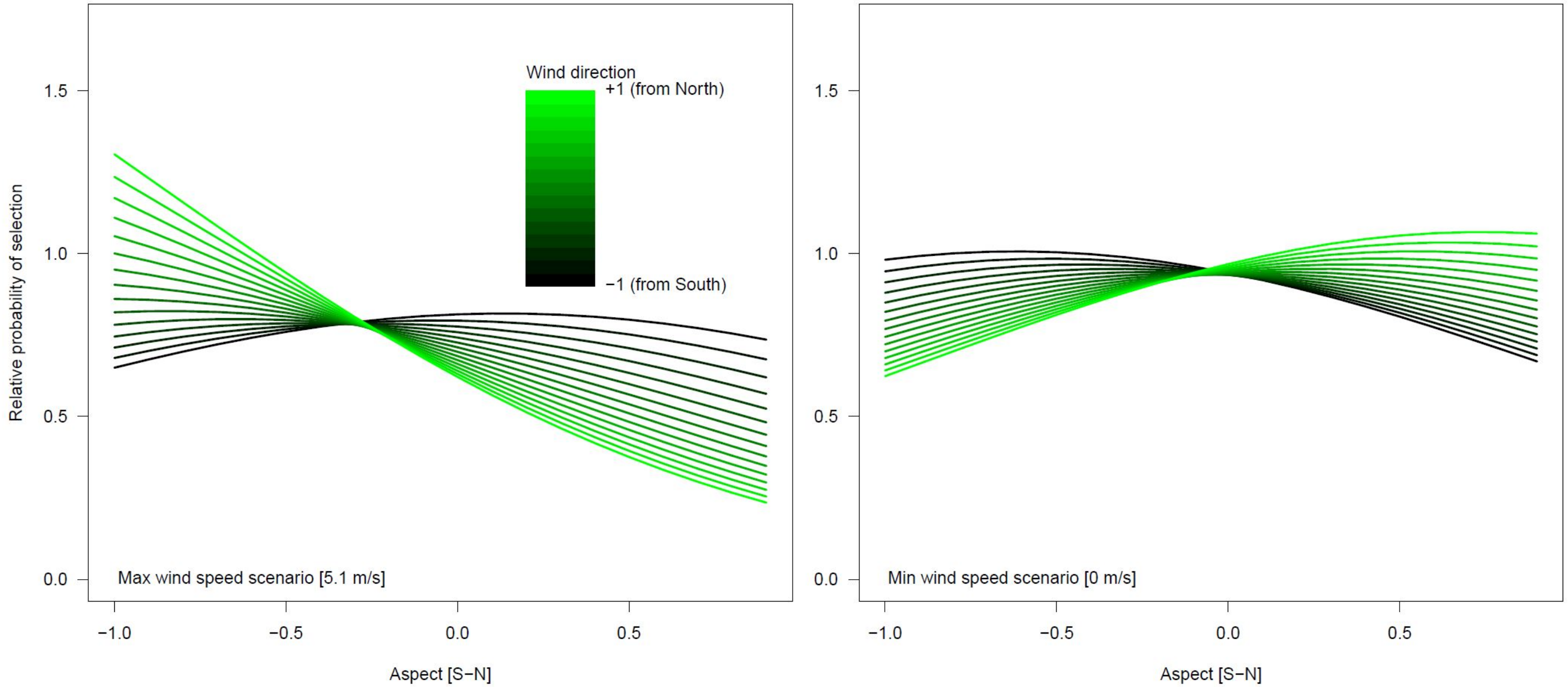

Figure S1.4 - Relative probability of selection for aspect (cos-transformed, x-axes) interacted with wind direction (different colours represent different scenarios for wind direction) and wind speed (the two plots represent two different scenarios) as predicted by the small-scale resource selection function, which was built using male ibex observations collected from May to October (2010-2011) in the Gran Paradiso National Park, Italy.

2 – Resource Selection Function k-fold validation

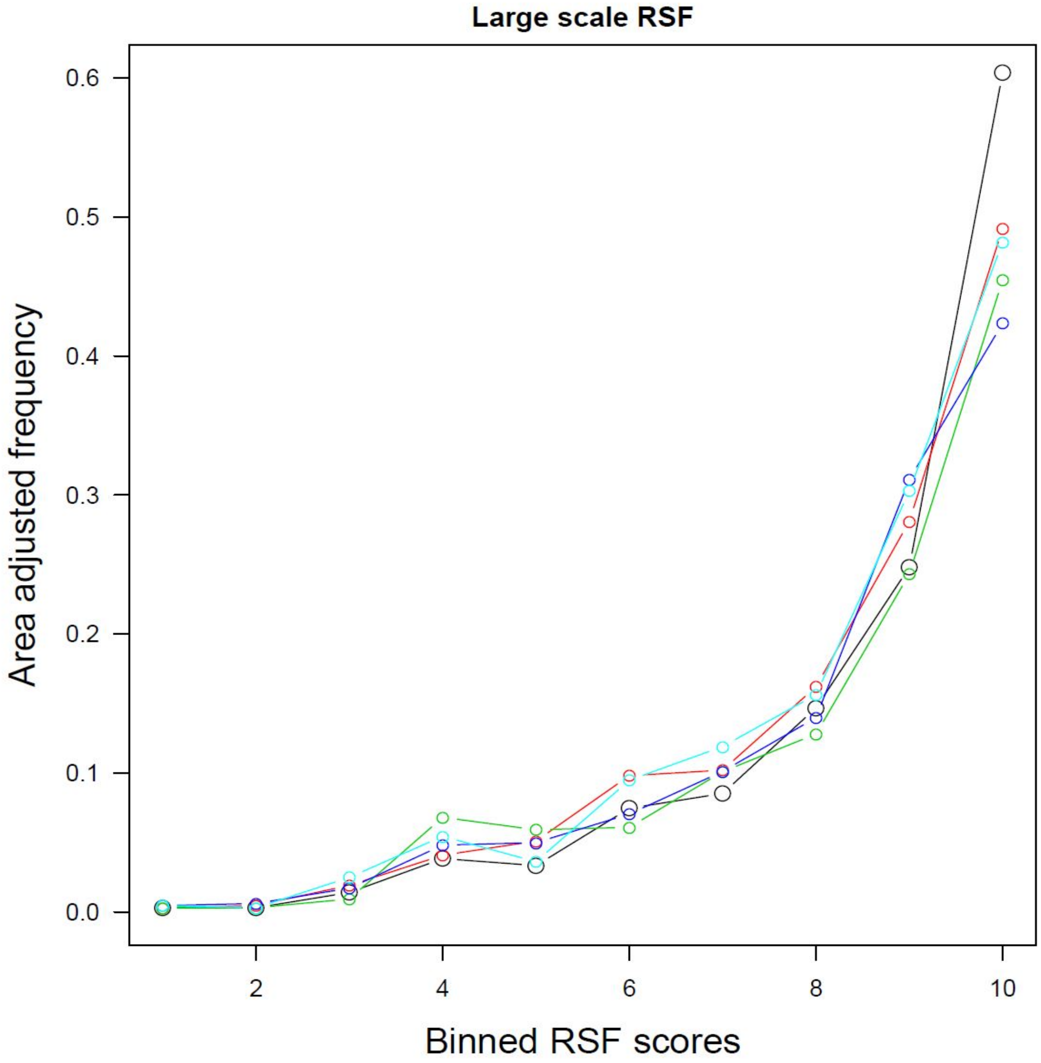

Figure S2.1 - Large-scale resource selection function (RSF) evaluation: area-adjusted frequency of categories (bins) of RSF scores. The evaluation implied calculating the correlation between RSF ranks and area-adjusted frequencies for a withheld sub-sample of data, e.g. 1/5 of the data in a 5-fold cross-validation scheme. We investigated the pattern of predicted RSF scores for partitioned testing data (presence-only) against categories of RSF scores (10 bins). A Spearman rank correlation between area-adjusted frequency of cross-validation points within individual bins and the bin rank was calculated for each cross-validated model. A model with good predictive performance would be expected to be one with a strong positive correlation, as more use locations (area-adjusted) would progressively fall into higher RSF bins. In this case, the 5-fold cross-validation showed that the large-scale resource selection model (Table 1 of the main manuscript) with daily maximum temperature as predictor had outstanding predictive ability on withheld data (Spearman correlation coefficients: $\rho_{fold1} = 0.988$, $\rho_{fold2} = 0.988$, $\rho_{fold3} = 0.976$, $\rho_{fold4} = 0.976$, $\rho_{fold5} = 0.988$).

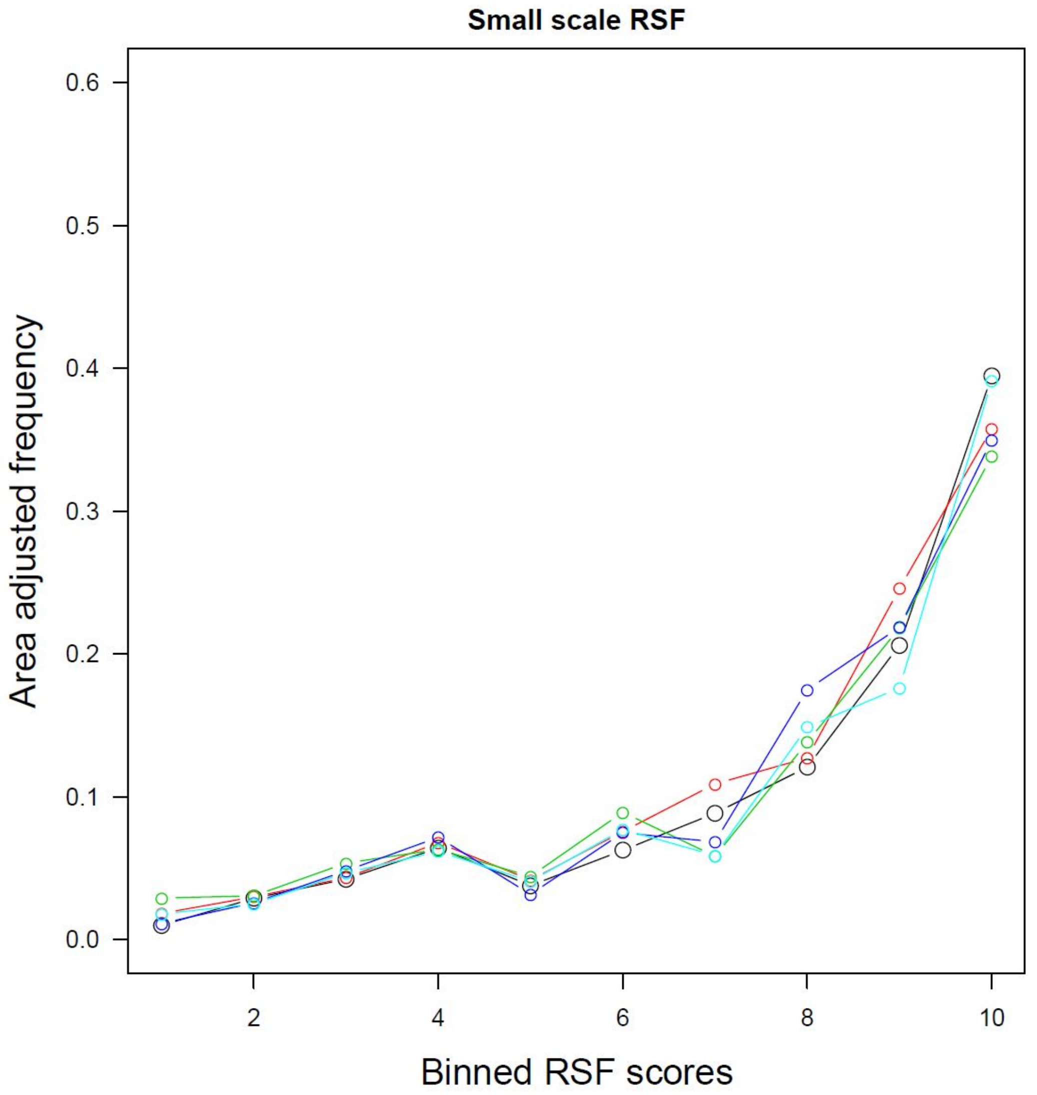

Figure S2.2 - Small-scale resource selection function (RSF) evaluation: area-adjusted frequency of categories (bins) of RSF scores. The evaluation implied calculating the correlation between RSF ranks and area-adjusted frequencies for a withheld sub-sample of data, e.g. 1/5 of the data in a 5-fold cross-validation scheme. We investigated the pattern of predicted RSF scores for partitioned testing data (presence-only) against categories of RSF scores (10 bins). A Spearman rank correlation between area-adjusted frequency of cross-validation points within individual bins and the bin rank was calculated for each cross-validated model. A model with good predictive performance would be expected to be one with a strong positive correlation, as more use locations (area-adjusted) would progressively fall into higher RSF bins. Compared to the large-scale resource selection model (Fig. S2.1), the small scale RSF had a weaker - but still very good - predictive ability on withheld data (ρ_{fold1} =0.881, ρ_{fold2} =0.912, ρ_{fold3} = 0.952, ρ_{fold4} =0.967, ρ_{fold5} =0.939).

3 - Large scale resource selection analysis: additional results

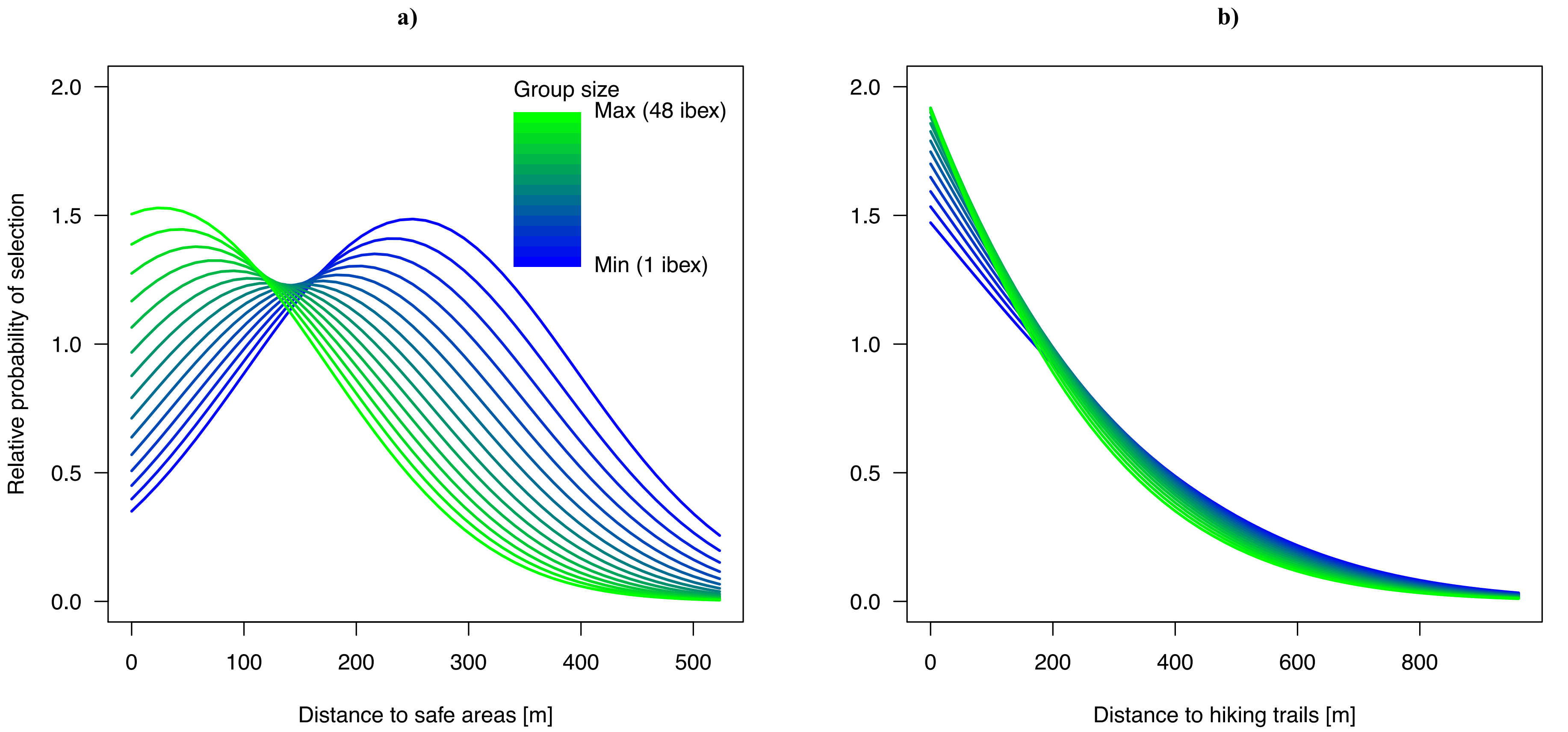

Figure S3.1 - Relative probability of selection for the distance to safe areas (a) and the distance to hiking trails (b), both interacted with ibex group size, as predicted by the large-scale resource selection function, which was built using male ibex observations collected from May to October (2010-2011) in the Gran Paradiso National Park, Italy.

4 – Resource Selection Function projections for RCP 4.5

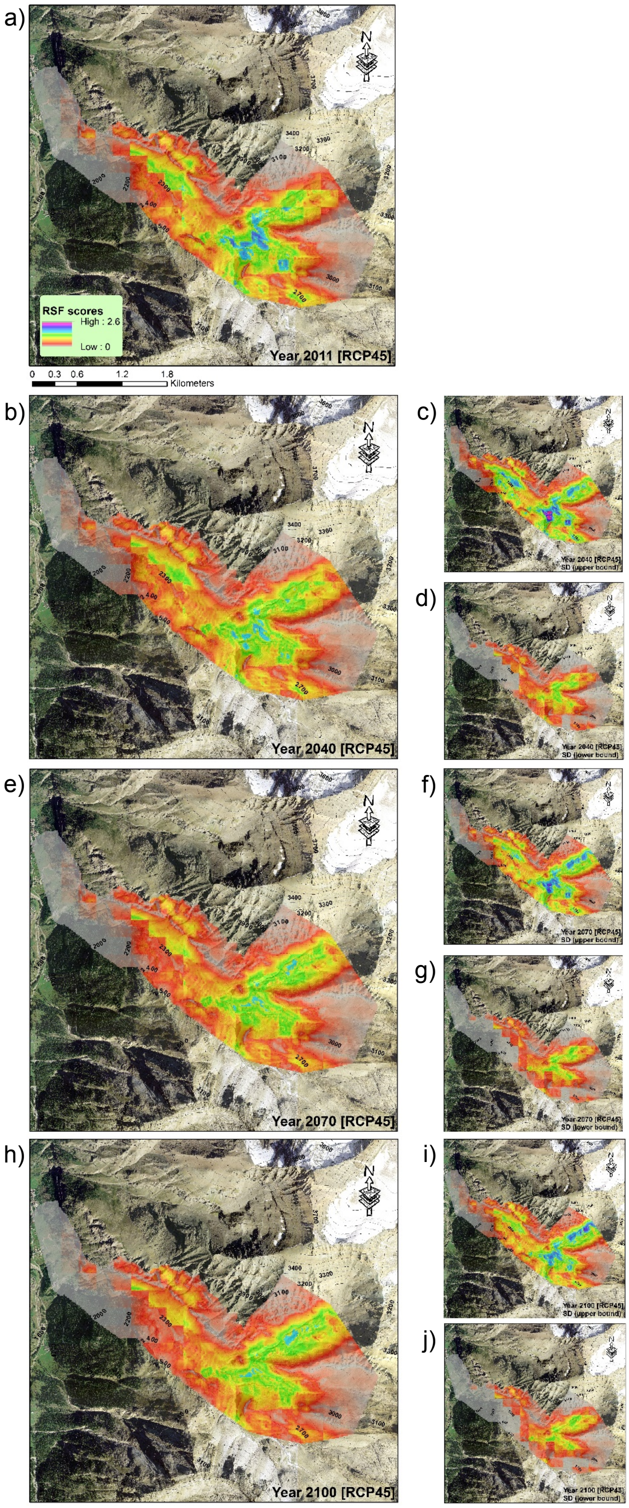

Figure S4.1 - Male ibex resource selection predicted in the Levionaz Valley, Gran Paradiso National Park, Italy, in 2011 (a, when this study was carried out) compared to years 2040, 2070, and 2100. Future scenarios are based on temperature projections forecasted by the RCP 4.5 climate models. Large plots depict the average RSF scores across RCP 4.5 simulations (b, e, h), whereas small plots represent upper (c, f, i) and lower standard deviation bounds (d, g, j), respectively. Maps were generated in ArcGIS 10.3 (ESRI 2011). Aerial imagery courtesy Gran Paradiso National Park, Italy.

5 – Additional maps and plots on data collection

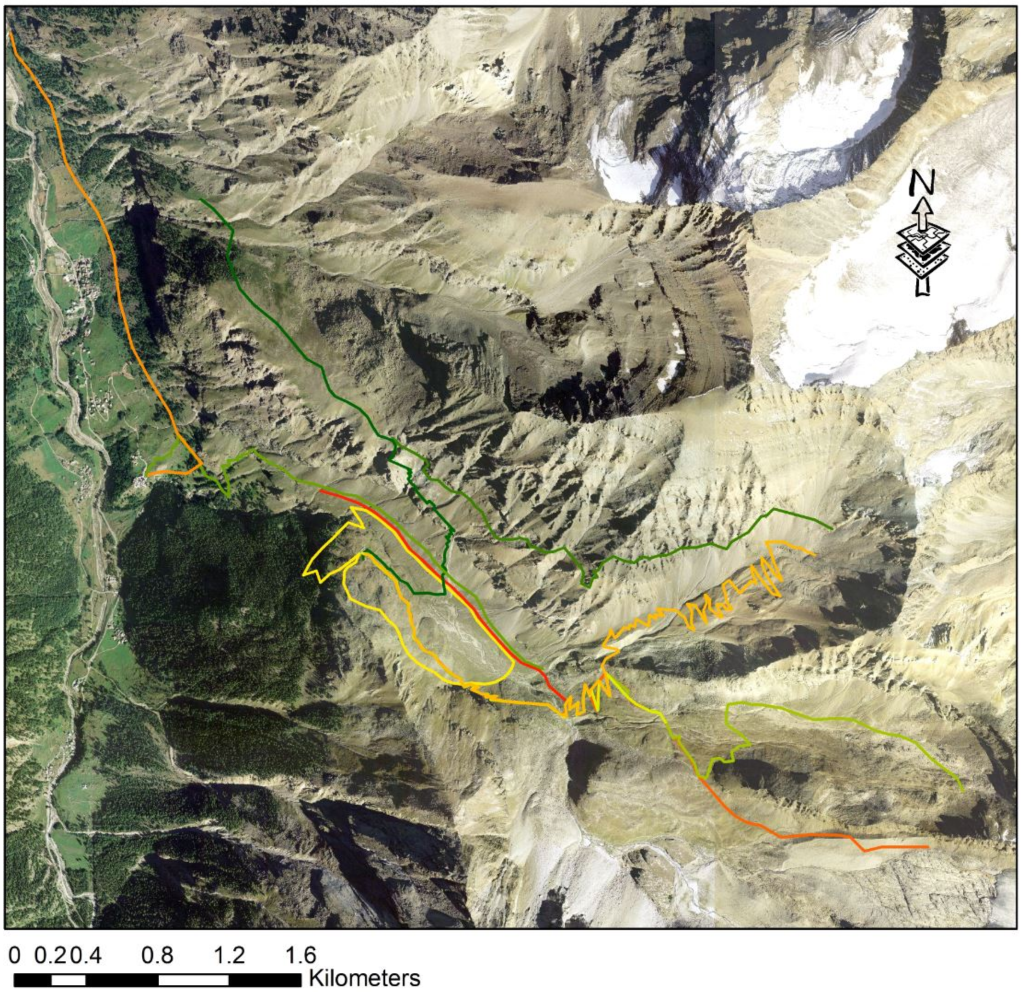

Figure S5.1 - Spatial distribution of the 10 hiking trails (colour-coded) used to locate male ibex from May to October (2010-2011) in the Levionaz valley, Gran Paradiso National Park, Italy. The map was generated in ArcGIS 10.3 (ESRI 2011). Aerial imagery courtesy Gran Paradiso National Park, Italy.

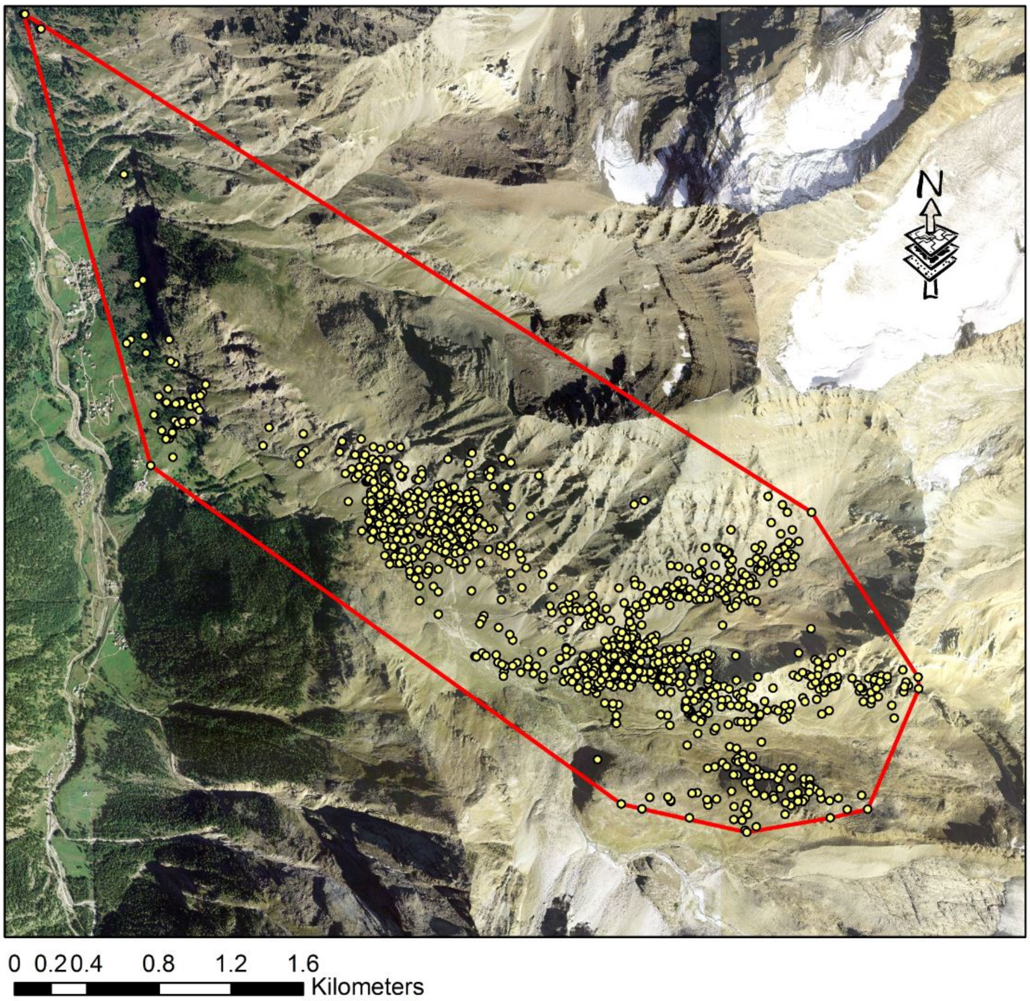

Figure S5.2 - Population-level Minimum Convex Polygon (MCP 100%) estimated by using relocations of individually-recognizable male ibex observed in 2010 and 2011 in the Levionaz valley, Gran Paradiso National Park, Italy. Because of the presence of outliers in the ibex spatial distribution, we did not add a buffer to the population-level home range, which otherwise is usual practice when depicting population-level home range in presence-availability studies. The map was generated in ArcGIS 10.3 (ESRI 2011). Aerial imagery courtesy Gran Paradiso National Park, Italy.

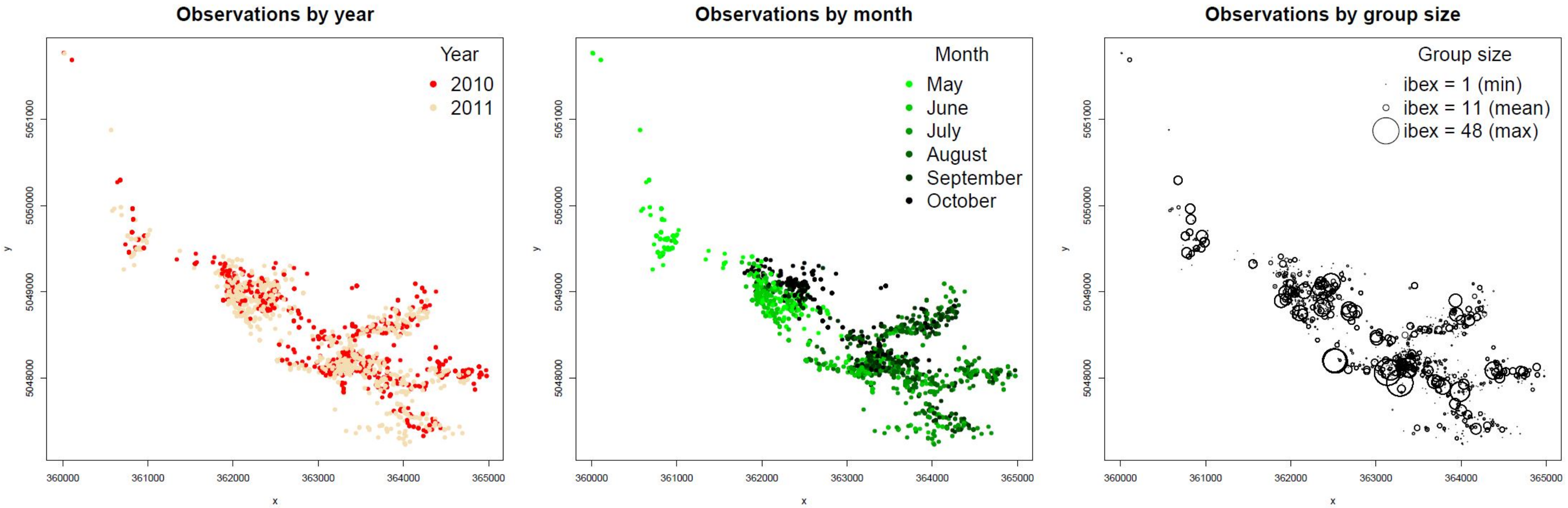

Figure S5.3 - Ibex observations carried out in the Levionaz valley (Gran Paradiso National Park, Italy) colour-coded by year (2010, 2011) and month of study (May through October), and group size (inclusive of solitary individuals). X and Y correspond to easting and northing, respectively, and the area depicted in each plot corresponds exactly to that depicted in Figs S5.1 and S5.2.

a)

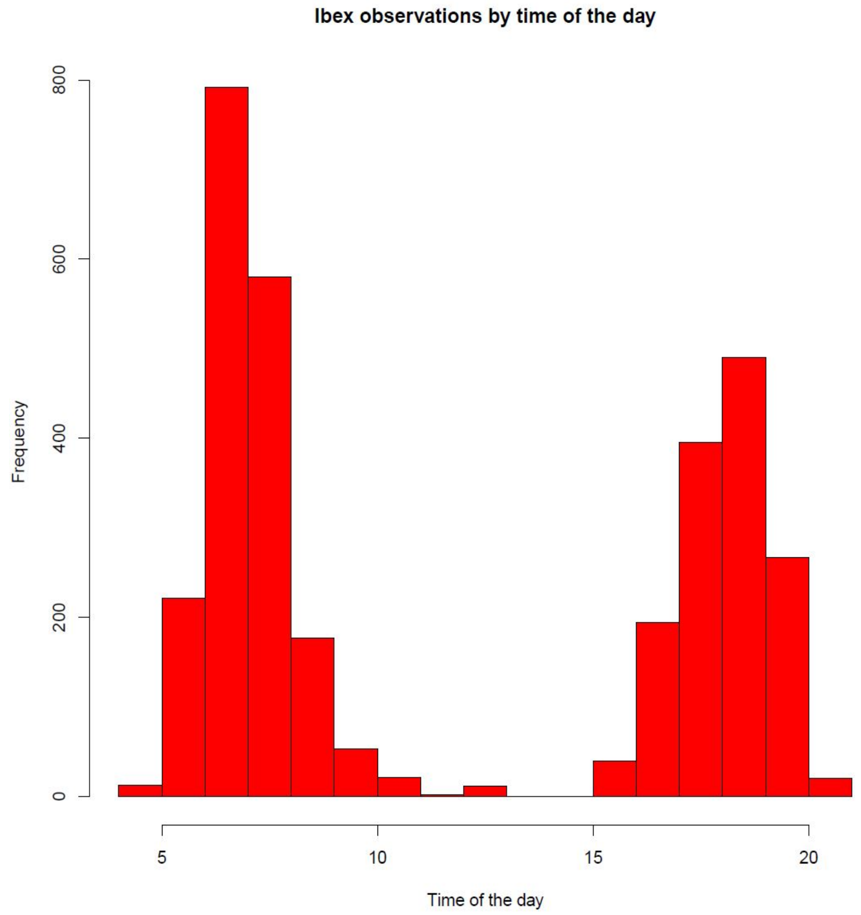

b)

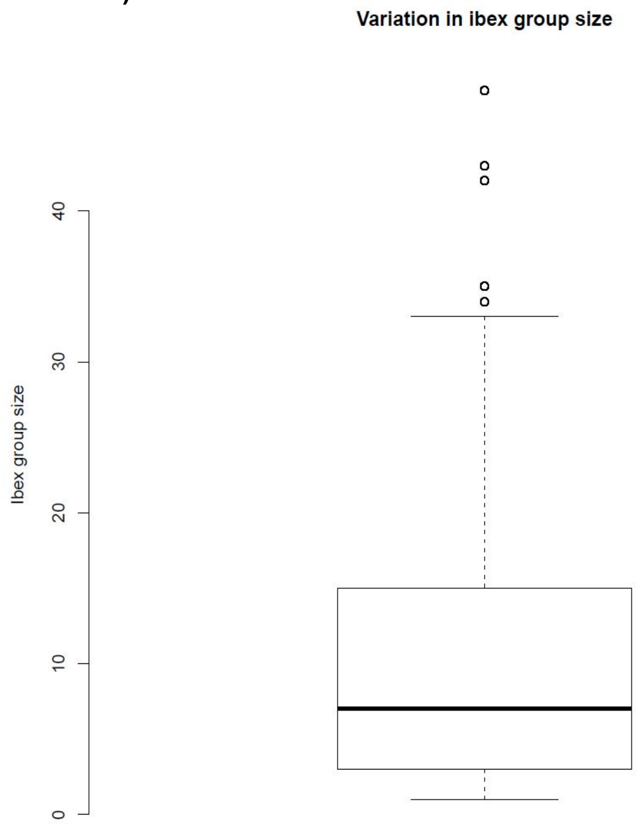

Figure S5.4 - Frequency of ibex observations as a function of time of the day (a) and observed variation in ibex group size (b). Direct observations were carried out from May through October (2010-2011) in the Levionaz valley, Gran Paradiso National Park, Italy.

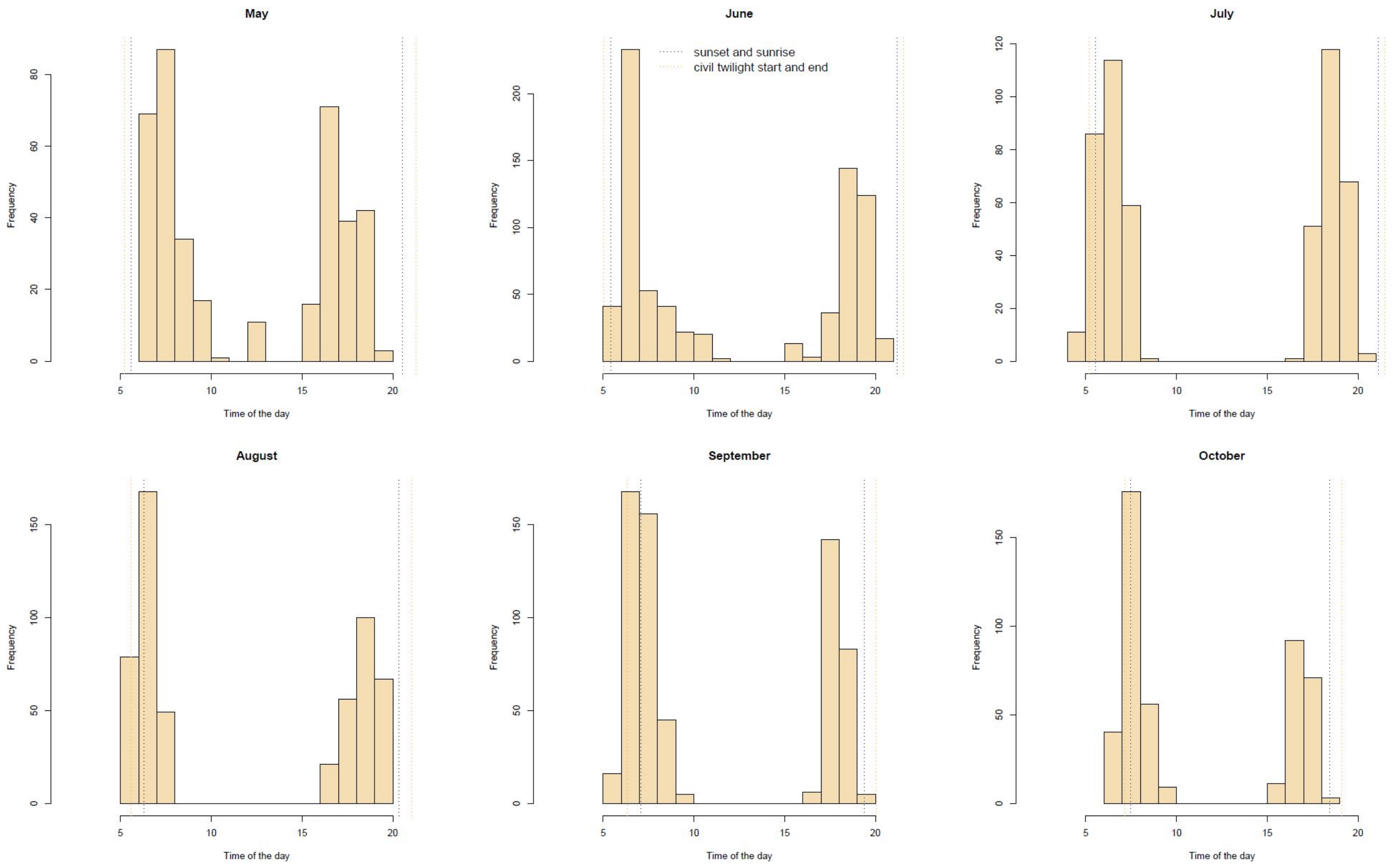

Figure S5.5 - Monthly frequency of ibex observations - as a function of time of the day - carried out from 2010 to 2011 in the Levionaz valley, Gran Paradiso National Park, Italy. Vertical dotted lines represent the time of sunset and sunrise as well as of the civil twilight start and end (see colour legend in the top-central plot)

6 - Temperature interpolation models

Background

We collected high-resolution temperature data in our study site and built interpolation models to predict fine-scale temperature variation over space and time during the monitoring period. We combined weather data collected at the nearest local weather station with temperature logger data (iButton DS1922L, Maxim Integrated) that we located throughout the study site over the study period. We had two different expectations about the effect of temperatures on ibex habitat selection: we hypothesised that ibex either select habitat based on the actual temperature (i.e., hourly temperature) - meaning that they are more likely to be located where the conditions are optimal in that moment - or based on the overall daily temperature (i.e., daily maximum temperature) - meaning that they are located at the elevation where the conditions will be optimal when the maximum temperature will be recorded. As a consequence, we built two temperature interpolation models predicting hourly temperature and daily maximum temperature, respectively.

Methods

Temperature loggers

We randomly distributed the temperature loggers (n = 15 in 2010, n = 17 in 2011; Fig. S6.1) in the meadows of the study site after stratifying by hydro-geographic sector (Fig. S6.2) and elevation (1 logger every 200 meters a.s.l.). Each logger was in the centre of a white cylindrical box at 1 m height from the ground, being the side facing the ground open. We programmed the loggers to collect air temperature every hour. We described below the two steps that were necessary to build the temperature interpolation models.

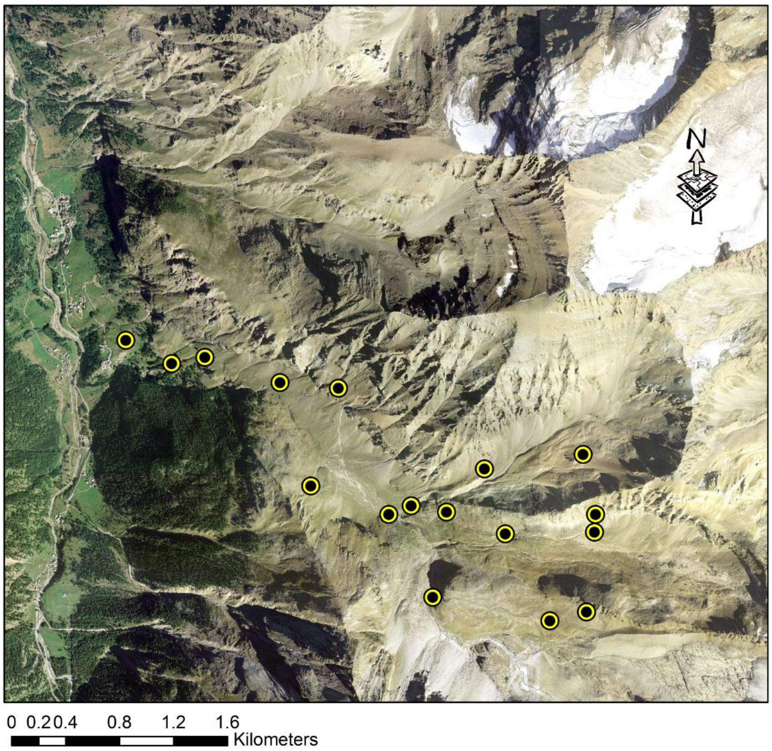

Figure S6.1 - Spatial distribution of the temperature loggers (iButton DS1922L, Maxim Integrated) in the Levionaz valley, Gran Paradiso National Park, Italy. The map was generated in ArcGIS 10.3 (ESRI 2011). Aerial imagery courtesy Gran Paradiso National Park, Italy.

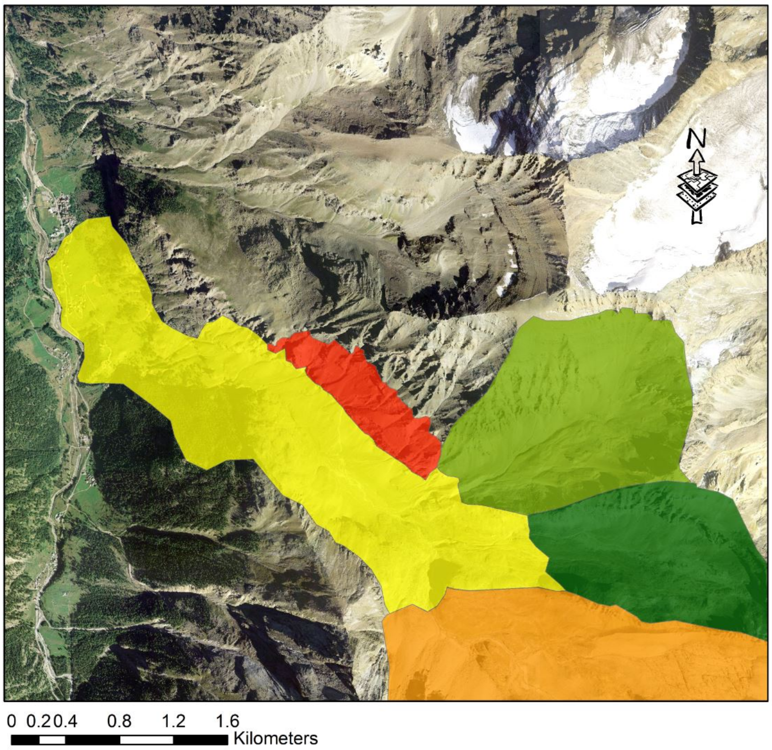

Figure S6.2 - Location of the hydro-geographic sectors - which differ in their micro-climate conditions - in the Levionaz valley, Gran Paradiso National Park, Italy. The map was generated in ArcGIS 10.3 (ESRI 2011). Aerial imagery courtesy Gran Paradiso National Park, Italy.

Step 1: filling in missing data in the dataset of the temperature loggers

Some of the loggers stopped recording temperature data, providing measurements for about 70-80% of the study period. For each data logger with missing data, we fitted linear models with air temperature as a response variable and the following covariates as predictors: time of the day (including quadratic and cubic term to account for nonlinearity), Julian day (including a quadratic term), year of study, their interactions (Julian date × time of the day, Julian date × year of study), and the air temperature recorded by the Pont weather station or by any other temperature logger. Regarding the latter predictor, we selected the temperature series that had the strongest correlation with the air temperature data collected by the target logger for which we wanted to replace missing data. Because of difference in altitude between the weather station or temperature loggers and the target logger, in some linear models we temporally shifted (1-3 hours) the response variable (temperature of the target logger) and the predictor (temperature recorded by the weather station or another logger selected based on the strength of the correlation) in order to achieve the best predictive model.

Once we built the starting linear model structure for each target logger, we then ran a forward and backward stepwise algorithm (*step* function of the *stats* package in R) and selected the best model structure with the lowest AIC values. We reported the adjusted R^2 as a measure of the predictive ability of each model, which we used to predict missing data and complete hourly temperature series of iButton loggers. After filling in all gaps, we calculated the daily maximum air temperatures.

Step 2: predicting hourly temperature and daily maximum temperature over space and time (interpolation models)

We used the logger temperature datasets to build spatial and temporal interpolation models and predict hourly temperatures for any 10 x 10 m pixel of the study site. We built a generalized additive model (GAM with Gaussian distribution of errors, *gam* function of the *mgcv* package) with hourly temperature as response variable and the following predictors: the Julian day (continuous variable fitted with smooth function), the time of the day (continuous variable fitted with smooth function), the year (categorical variable), the hydro-geographic sectors (categorical variable), the elevation where the temperature was recorded (continuous variable, 10 x 10 m spatial resolution, see below for the details), and the interaction between the time of the day and the Julian day, and between the Julian day and the year of study. After screening the data, we decided to fit two alternative model structures to make sure taking into account the effect of the elevation properly: one model with elevation fitted with the smoothing function of the GAM, the other one with elevation without smoothing function but rather included as simple linear and quadratic term.

We repeated the same procedure and built two alternative GAMs to model daily maximum temperatures. We used the same set of predictors as for the hourly temperature models, this time

removing the time of the day and its interactions because meaningless when modelling the daily maximum temperature.

We random-cross-validated our alternative models to verify their ability to predict on new data. We trained the best model on 80% of the data, predicted on the remaining 20%, and computed the R^2 of the relationship between predicted and observed temperature data. We extracted the average R^2 after repeating the procedure 50 times.

Results

Step 1: filling in missing data in the dataset of the temperature loggers

Best linear models used to fill in missing data of temperature loggers were reported in Table S6.1. We used the related model equations to predict air temperatures and replace missing data in the logger temperature time series.

Table S6.1 - Structures and performances of models used to fill in missing values in the temperature logger (iButton) datasets deployed in the Levionaz study area,Gran Paradiso National Park, Italy.

I-button ID	time shift, if needed	Model predictors									adj. R^2
		Temp recorded by the weather station or another logger (ID)	year	Julian day	Julian day^2	Time of the day	Time of the day^2	Time of the day^3	Julian day × time of the day	Julian day × year	
1	no	ibutton (2)	yes	yes	yes	yes	yes	yes	yes	yes	0.945
2	3 hours	weather station	yes	yes	yes	yes	yes	yes	yes	yes	0.935
3	3 hours	weather station	yes	yes	yes	yes	yes	yes	no	yes	0.933
4	3 hours	weather station	yes	yes	yes	yes	yes	yes	yes	yes	0.922
5	no	ibutton (8)	no	yes	yes	yes	yes	yes	yes	no	0.922
6	2 hours	weather station	yes	yes	yes	yes	yes	yes	no	no	0.937
7	2 hours	ibutton (6)	yes	yes	no	yes	yes	yes	yes	yes	0.820
8	2 hours	weather station	yes	yes	yes	yes	yes	yes	yes	yes	0.910
9	no	ibutton (8)	yes	yes	yes	no	yes	yes	no	yes	0.947
10	no	ibutton (12)	no	yes	yes	yes	yes	yes	yes	no	0.946
11	2 hours	ibutton (6)	yes	yes	yes	yes	yes	yes	yes	yes	0.950
12	2 hours	weather station	yes	yes	yes	yes	yes	no	yes	no	0.900
13	no	ibutton (12)	yes	yes	yes	yes	yes	yes	yes	yes	0.912
14	no	ibutton (12)	yes	yes	yes	yes	yes	yes	yes	yes	0.911
15	no	ibutton (8)	yes	yes	yes	yes	yes	yes	no	yes	0.951
16	no	ibutton (6)	no	yes	yes	yes	yes	yes	no	no	0.778
17	1 hour	ibutton (4)	no	yes	no	yes	yes	yes	no	no	0.929

Step 2: predicting hourly temperature and daily maximum temperature over space and time (interpolation models)

We reported the two alternative models explaining the variability of hourly temperatures in Table S6.2. The model with elevation fitted as smooth term in the GAM outperformed the alternative model (with elevation fitted as linear and quadratic term) and was used to predict hourly temperature over space and time during the study period.

Table S6.2 - Alternative Generalized Additive Models (GAMs) built to interpolate hourly temperatures over space and time in the Levionaz Valley, Gran Paradiso National Park [β_i refers to parameters estimated for predictors fit as a linear model, whereas s_i refers to smoothing functions of the generalized additive component of the model; Δ AIC= difference in Akaike Information Criterion between the best and the alternative model; μR^2 = mean of R^2 values calculated by cross validation - see text for more details). The model selected for final interpolations included elevation fitted as smoothing spline.

Model structure	ΔAIC	μR^2
$y = \beta_0 + \beta_1 sector + \beta_2 year + s_1(elevation) + s_2(time) + s_3(julian\_date,\ time) + s_4(julian\_date,\ by = year)$	0	0.69
$y = \beta_0 + \beta_1 sector + \beta_2 year + \beta_3 elevation + \beta_4 elevation\hat{}2 + s_1(time) + s_2(julian\_date,\ time) + s_3(julian\_date,\ by = year)$	1057.2	0.66

Likewise, we reported the two alternative models explaining the variability in daily maximum temperatures in Table S6.3. Also in this case, the model with elevation fitted as smooth term in the GAM outperformed the alternative model (with elevation fitted as linear and quadratic term) and was used to predict daily maximum temperature over space and time during the study period.

Table S6.3 - Alternative Generalized Additive Models GAMs built to interpolate daily maximum temperatures over space and time in the Levionaz Valley, Gran Paradiso National Park [β_i refers to parameters estimated for predictors fit as a linear model, whereas s_i refers to smoothing functions of the generalized additive component of the model; Δ AIC= difference in Akaike Information Criterion between the best and the alternative model; μR^2 = mean of R^2 values calculated by cross validation - see text for more details). The model selected for final interpolations included elevation fitted as smoothing spline.

Model structure	ΔAIC	μR^2
$y = \beta_0 + \beta_1 sector + \beta_2 year + s_1(elevation) + s_2(julian, by = year)$	0	0.589
$y = \beta_0 + \beta_1 sector + \beta_2 year + \beta_3 elevation + \beta_4 elevation\hat{}2 + s_1(julian, by = year)$	972.1	0.514

7 - Calculation of buffer sizes needed to depict random availability in small-scale resource selection analysis.

To define the buffer size for small-scale resource selection analysis (i.e., the area around each ibex presence location where to depict random availability), we made use of satellite telemetry data. We screened telemetry data available for male ibex in the Levionaz valley (year 2013), and we estimated ibex monthly mobility. Such movement behaviour can give us a clue on how far an ibex can move monthly, and thus define where to set the limit for sampling available resources in small-scale resource selection analysis. Because we used telemetry data collected in 2013 to define small-scale availability for ibex observed in 2010 and 2011, we decided to keep a more conservative approach and use a monthly temporal scale rather than depicting buffer sizes daily. The latter approach might have been biased by different environmental conditions occurring in the two different study periods.

Telemetry data were available for 10 male ibex fitted with Vectronic GPS collars (GPS PRO Light collar, Vectronic Aerospace GmbH) with 7-h relocation schedule. See Table S7.1 for details on sampling.

Table S7.1 - Sample size of satellite telemetry relocations recorded for n = 10 collared male ibex from early May to late October 2013 in the Levionaz valley,Gran Paradiso National Park.

GPS-collar ID	Age of the Male (y.o.)	Total number of satellite relocations					
		May	Jun	Jul	Aug	Sep	Oct
12227	8		141	54			
12228	9	94	111	95	96	100	96
12229	13	99	90	91	86	90	
12230	9		27	154	4		
12231	9	95	114	87			
12232	9	110	96	50			
12233	11		26	294	95	96	78
12234	9	131	93	95	4		
12235	8		134	96	98	99	95
12236	11	133	95	101	98	100	99
Sample size	-	6	10	10	7	5	4
Average	9.6	110.3	92.7	111.7	68.7	97.0	92.0
Total	-	662	927	1117	481	485	368

We computed for each individual ibex the distances between successive locations covered monthly and extracted the 75% quantile. We used the 75%-quantile rather than the maximum distance to avoid bias from relocation outliers (*sensu* Duchesne et al.[1]). We averaged monthly the quantiles extracted for all ibex (Table S7.2), thus defining the maximum distance that an ibex may cover every month. These distances were used as radiuses for buffers that were eventually used to identify the circular area

around each ibex relocation where to draw the random availability for small-scale resource selection analysis.

Table S7.2 - Radius of the buffers (in meters) defining monthly availability around each ibex observations to be used in small-scale resource selection analysis.

May	June	July	August	September	October
400.2	1358.1	1363.8	785.7	1179.5	1016.2

8 - Sensitivity analysis aimed at defining the minimum number of random available points to be associated with ibex presence data in resource selection analyses.

Aim of this supplementary information is to run a sensitivity analysis and define the minimum number of available points that need to be associated to ibex presence data in resource selection functions.

Methods

Following recommendations by Ciuti et al.[3] and Roberts et al.[4] (see box 2 therein), we fit a generalized linear mixed-effect model with Bernoulli distribution of errors, presence (1) and availability (0) as response variable, and air temperature predicted by interpolation models as predictor. We used air temperature because it was the environmental covariate collected at the finest spatial resolution (10 x 10 m). We fitted the stratum-ID (identifying each pair of used location with its associated random available locations) nested within the individual-ID (identifying the individually recognizable ibex) as random intercept in the model; we also fitted the group-ID (identifying the identity of the group where the marked ibex was observed) as (crossed) random intercept.

We started the sensitivity analysis with an available:used location ratio 1:1 by running the GLMM 10 times, each time drawing a new random spatial sample of available locations. We repeated this procedure several times, stepwise increasing the number of random available points associated with each used point (i.e., available:used 2:1, 3:1, …, 50:1), and extracted the estimated model parameters (beta estimates). We thus screened the variation of beta estimates as a function of the number of random available locations, and fit a generalized additive model (response: beta estimates; predictor: number of random available locations per used location) to describe the relationship and detect model parameter stabilisation. Based on the visual inspection of GAMs, we selected the thresholds for the minimum number of random points needed to get stable parameter estimates.

Results

Results of the sensitivity analyses for large-scale and small-scale analyses are reported in Figs. S8.1-4. We selected 15 random points per used point in the large-scale resource selection analysis, whereas we selected 13 random points per used point in the small-scale analysis.

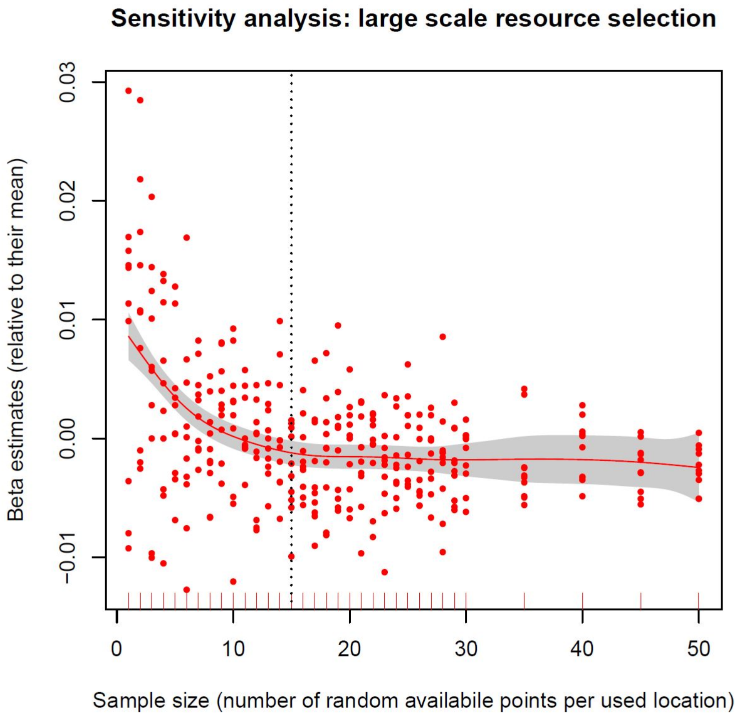

Figure S8.1 - General Additive Model (GAM) depicting the trend of the parameter estimates for air temperature in GLMM (large-scale analysis) with varying sample size of random available points per used location. The vertical dashed line indicates the turning point when beta estimates get stable.

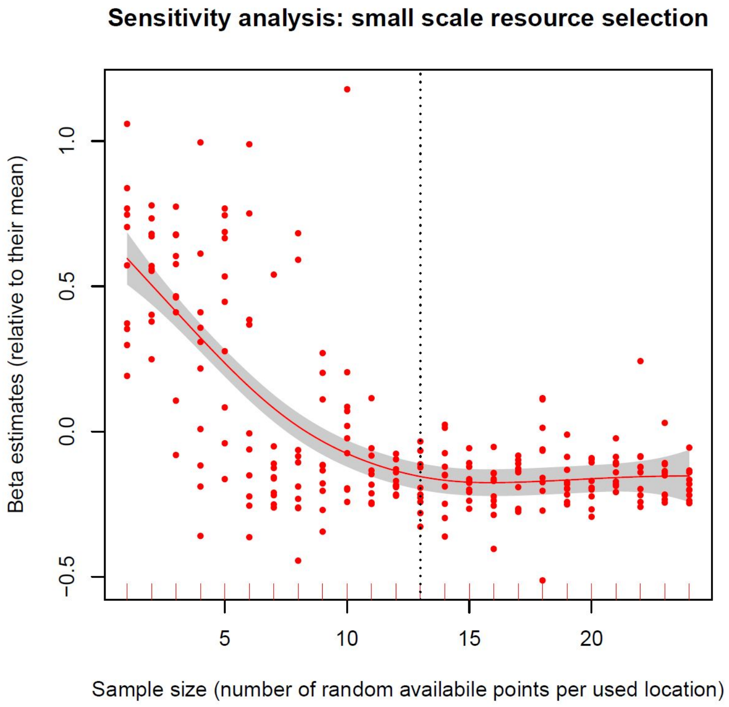

Figure S8.2 - General Additive Model (GAM) depicting the trend of the parameter estimates for air temperature in GLMM (small-scale analysis) with varying sample size of random available points per used location. The vertical dashed line indicates the turning point when beta estimates get stable.

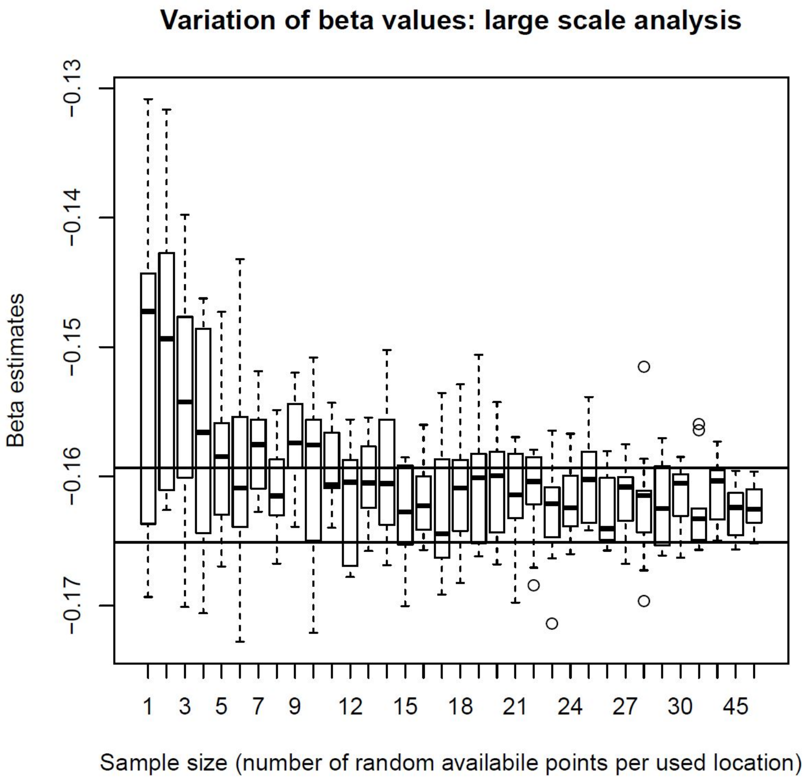

Figure S8.3 - Variation of the parameter estimates for air temperature in GLMM (large scale analysis) as a function of the varying sample size of random available points per used location.

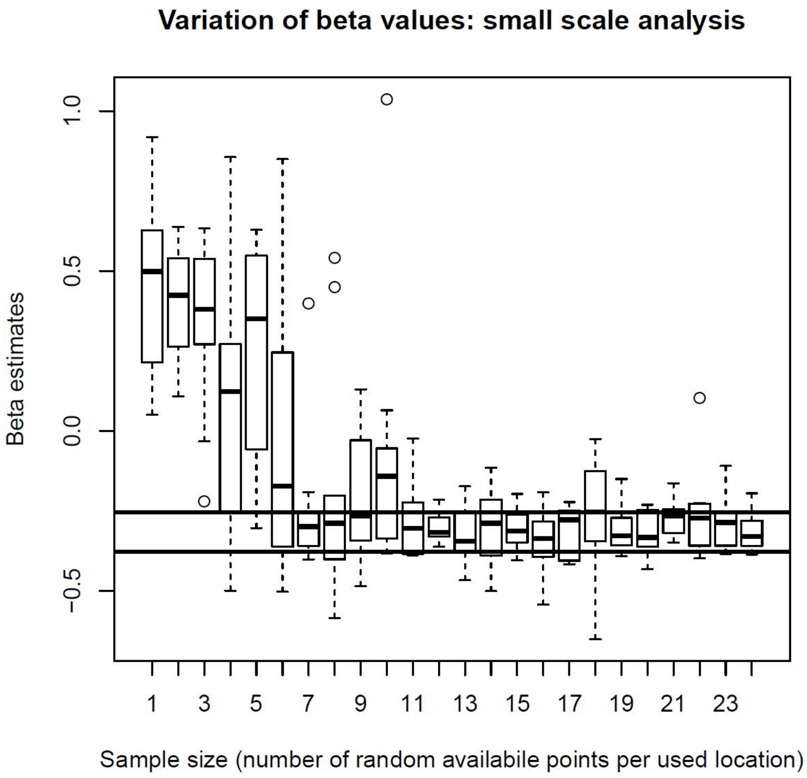

Figure S8.4 - Variation of the parameter estimates for air temperature in GLMM (small-scale analysis) as a function of the varying sample size of random available points per used location.

9 - Environmental covariates used in the ibex resource selection analyses

Table S9.1 - List of the covariates expected to drive ibex resource selection, which were included in the full model structure for both large- scale and small-scale resource selection by male ibex observed from 2010 to 2011 in the Gran Paradiso National Park, Italy. Variables included in the best models of both spatial scales are indicated in bold.

Type	Name	Description
Biological factors	**IDENTITY**	Identity of each marked male observed
	AGE	Age of each marked male observed
	**GROUP SIZE**	Size of the group where the ibex was observed
Temporal parameters	**JULIAN DATE**	Day of the year when the ibex were observed
	MONTH	Month of the year when the ibex were observed
	16-DAY PERIOD	16-days period (corresponding to the NDVI sampling rate)
	TIME OF THE DAY	Time of the day when the ibex were observed
	PART OF THE DAY	Part of the day when the ibex were observed, at three level: DAWN, DAY, DUSK
Weather	**AIR TEMPERATURE (HOURLY AND DAILY MAXIMUM)**	Data from temperature loggers were combined with those collected by the weather station and used to build interpolation models predicting hourly and maximum daily temperature (°C) for each 10 x 10 m pixel of the study area at any given day within the study period
	RADIATION	Solar radiation (W/m^2) recorded at the weather station
	**WIND_SPEED**	Speed of the wind (m/s) recorded at the weather station.
	**WIND_DIRECTION**	Direction of the wind recorded at the weather station, cosine-transformed to range between -1 with wind blowing from the South and +1 with wind blowing from the North.
Terrain	DEM (ELEVATION)	Digital Elevation Model (m)
	**ASPECT**	Terrain aspect, cosine-transformed (N-S)
	**SLOPE**	Degrees rise of the terrain
	TRI	Terrain Ruggedness Index (m) calculated based on the DOCELL code developed by Riley et al. (1999)
Forage quality	**NDVI**	Normalized Difference Vegetation Index (16-days-composite at 250 x 250 m pixel size)
Land cover	MEADOWS AND GRASSLAND	Meadows, meadows/pasture, grassland
	WOODS AND BUSHES	Larch and Swiss stone pine woods, pioneer woods, invasive bushes, bushes
	SCREES AND ROCKS	Rocks, screes, river banks
	OTHERS	Abandoned crop fields, urban areas/infrastructure
Predation risk	**DIST_SAFE_AREAS_45**	Distance to safe areas defined by a slope > 45 (m)
	DIST_SAFE_AREAS_30	Distance to safe areas defined by a slope > 30 (m)
	**DIST_HIKING_TRAILS**	Distance to hiking trails (m), log-transformed.
	NUMBER_HIKERS	Estimate of the average number of hikers using the trails (GPNP, official data).

10 - Additional information on climate models.

Table S10.1 - Full details on the CMIP5 models used in this study, ordered by meridional resolution. The RCP column indicates whether data for the RCP 4.5 (4) or for the RCP 8.5 (8) scenario were available. Thirty and 38 scenarios were eventually available for RCP 8.5 and RCP 4.5, respectively.

Model ID	Institution ID	Resolution lon × lat[°] - Levels	RCP	Key references
CMCC-CM	CMCC	0.75 × 0.75L31 (T159)	4,8	Scoccimarro et al. 2011[4]
CCSM4	NCAR	1.25 × 0.9L27 (T63)	4,8	Meehl et al. 2012[5]
CESM1-BGC	NSF-DOE-NCAR	1.25 × 0.9L27	4,8	Hurrell et al. 2013[6]
CESM1-CAM5	NSF-DOE-NCAR	1.25 × 0.9L27	4,8	Hurrell et al. 2013[6]
bcc-csm1-1-m	BCC	1.125 × 1.125L26 (T106)	4	Wu et al. 2013[7]
EC-EARTH	EC-EARTH	1.125 × 1.125L62 (T159)	4,8	Hazeleger et al. 2012[8]
MRI-CGCM3	MRI	1.125 × 1.125L48 (T159)	4,8	Yukimoto et al. 2012[9]
CNRM-CM5	CNRM-CERFACS	1.40625 × 1.40625L31 (T127)	4,8	Voldoire et al. 2013[10]
MIROC5	MIROC	1.40625 × 1.40625L40 (T85)	4,8	Watanabe et al. 2010[11]
ACCESS1-0	CSIRO-BOM	1.875 × 1.25L38 (N96)	4,8	Bi et al. 2013[12]
ACCESS1-3	CSIRO-BOM	1.875 × 1.25L38 (N96)	4,8	Bi et al. 2013[12]
HadGEM2-AO	MOHC	1.875 × 1.24L60 (N96)	4,8	Martin et al. 2011[13]
HadGEM2-CC	MOHC	1.875 × 1.24L60 (N96)	4,8	Martin et al. 2011[13]
HadGEM2-ES	MOHC	1.875 × 1.24L60 (N96)	4,8	Martin et al. 2011[13]
MPI-ESM-LR	MPI	1.875 × 1.875L47 (T63)	4,8	Giorgetta et al. 2013[14]
MPI-ESM-MR	MPI	1.875 × 1.875L95 (T63)	4,8	Giorgetta et al. 2013[14]
IPSL-CM5A-MR	IPSL	2.5 × 1.2587L39	4,8	Hourdin et al. 2013[15]
INM-CM4	INM	2 × 1.5L21	4	Volodin et al. 2010[16]
CSIRO-Mk3-6-0	CSIRO-QCCCE	1.875 × 1.875L18 (T63)	4,8	Rotstayn et al. 2012[17]
NorESM1-M	NCC	2.5 × 1.9L26 (F19)	4,8	Bentsen et al. 2013[18]
GFDL-CM3	GFDL	2.5 × 2L48 (C48)	4,8	Delworth et al. 2006[19]
GFDL-ESM2G	GFDL	2.5 × 2L24 (M45)	4,8	Delworth et al. 2006[19]
GFDL-ESM2M	GFDL	2.5 × 2L24 (M45)	4,8	Delworth et al. 2006[19]
GISS-E2-H	NASA/GISS	2.5 × 2L24	4	Schmidt et al. 2006[20]
GISS-E2-R	NASA/GISS	2.5 × 2L24	4	Schmidt et al. 2006[20]
GISS-E2-H-CC	NASA/GISS	2.5 × 2L24	4	Schmidt et al. 2006[20]
GISS-E2-R-CC	NASA/GISS	2.5 × 2L24	4	Schmidt et al. 2006[20]
IPSL-CM5A-LR	IPSL	3.75 × 1.89L39	4,8	Hourdin et al. 2013[15]
IPSL-CM5B-LR	IPSL	3.75 × 1.9L39	4,8	Hourdin et al. 2013[15]
HADCM3	MOHC	3.75 × 2.5L19 (N48)	4	Collins et al. 2011[21]
FIO-ESM	FIO	2.8125 × 2.8125L80 (T42)	4	Qiao et al. 2013[22]
MIROC-ESM-CHEM	MIROC	2.8125 × 2.8125L80 (T42)	4,8	Watanabe et al. 2011[11]
MIROC-ESM	MIROC	2.8125 × 2.8125L80 (T42)	4,8	Watanabe et al. 2011[11]
bcc-csm1-1	BCC	2.8125 × 2.8125L26 (T42)	4,8	Wu et al. 2013[7]
BNU-ESM	GCESS-BNU	2.8125 × 2.8125L26 (T42)	4,8	Ji et al. 2014[23]
CanESM2	CCCMA	2.8125 × 2.8125L35 (T63)	4,8	Arora et al. 2011[24]
FGOALS-g2	LASG-CESS	2.8125 × 2.8125L26	4,8	Li et al. 2013[25]
CMCC-CMS	CMCC	3.75 × 3.75L95 (T63)	4,8	Scoccimarro et al. 2011[4]

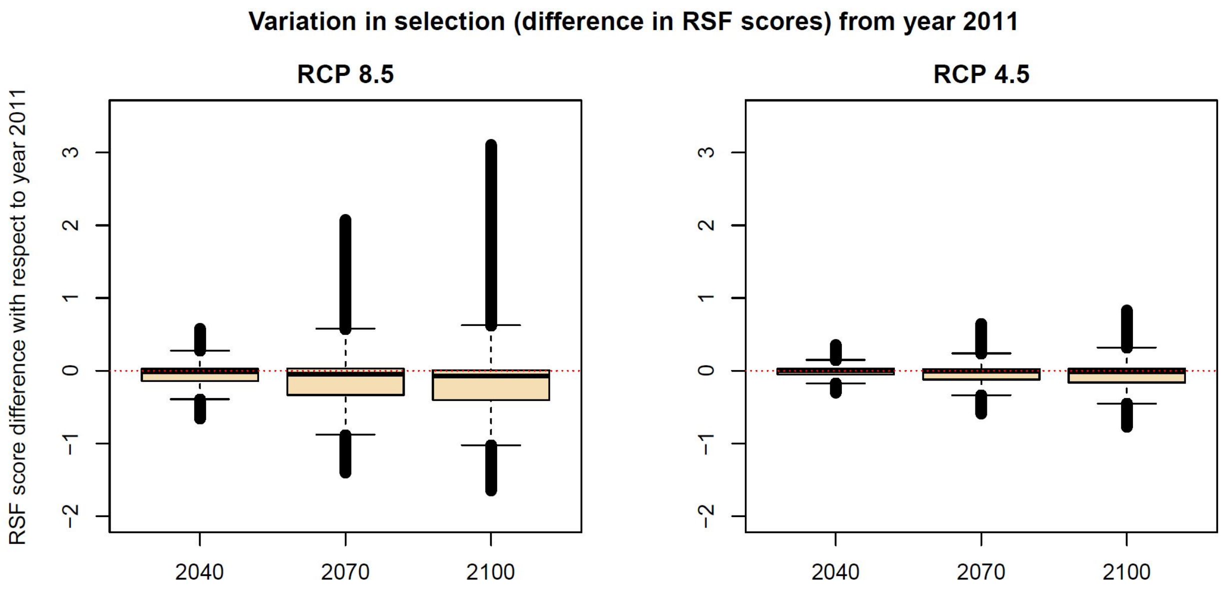

Figure S10.1 - Difference in RSF scores between those predicted for years 2040, 2070, and 2100 and those recorded for year 2011 (left panel, RCP 8.5 climate change scenario; right panel, RCP 4.5).

References

1. Duchesne, T., Fortin, D. & Courbin, N. Mixed conditional logistic regression for habitat selection studies. *J. Anim. Ecol.* **79,** 548–555 (2010).
2. Ciuti, S. *et al.* An efficient method to exploit LiDAR data in animal ecology. *Methods Ecol. Evol.* **9,** 893-904 (2018).
3. Roberts, D. R. *et al.* Cross-validation strategies for data with temporal, spatial, hierarchical, or phylogenetic structure. *Ecography* **40,** 913–929 (2017).
4. Scoccimarro, E. *et al.* Effects of Tropical Cyclones on Ocean Heat Transport in a High-Resolution Coupled General Circulation Model. *J. Clim.* **24,** 4368–4384 (2011).
5. Meehl, G. A. *et al.* Climate System Response to External Forcings and Climate Change Projections in CCSM4. *J. Clim.* **25,** 3661–3683 (2012).
6. Hurrell, J. W. *et al.* The Community Earth System Model: A Framework for Collaborative Research. *Bull. Am. Meteorol. Soc.* **94,** 1339–1360 (2013).
7. Wu, T. *et al.* Global carbon budgets simulated by the Beijing Climate Center Climate System Model for the last century: GLOBAL CARBON CYCLE FROM A CMIP5 MODEL. *J. Geophys. Res. Atmospheres* **118,** 4326–4347 (2013).
8. Hazeleger, W. *et al.* EC-Earth V2.2: description and validation of a new seamless earth system prediction model. *Clim. Dyn.* **39,** 2611–2629 (2012).
9. Yukimoto, S. *et al.* A new global climate model of the Meteorological Research Institute: MRI-CGCM3: model description and basic performance (special issue on recent development on climate models and future climate projections). *J. Meteorol. Soc. Jpn.* **90A,** 23–64 (2012).
10. Voldoire, A. *et al.* The CNRM-CM5.1 global climate model: description and basic evaluation. *Clim. Dyn.* **40,** 2091–2121 (2013).
11. Watanabe, M. *et al.* Improved Climate Simulation by MIROC5: Mean States, Variability, and Climate Sensitivity. *J. Clim.* **23,** 6312–6335 (2010).
12. Bi, D. *et al.* The ACCESS coupled model: description, control climate and evaluation. *Aust. Meteorol. Oceanogr. J.* **63,** 41–64 (2013).
13. The HadGEM2 Development Team: G. M. Martin *et al.* The HadGEM2 family of Met Office Unified Model climate configurations. *Geosci. Model Dev.* **4,** 723–757 (2011).
14. Giorgetta, M. A. *et al.* Climate and carbon cycle changes from 1850 to 2100 in MPI-ESM simulations for the Coupled Model Intercomparison Project phase 5: Climate Changes in MPI-ESM. *J. Adv. Model. Earth Syst.* **5,** 572–597 (2013).
15. Hourdin, F. *et al.* Impact of the LMDZ atmospheric grid configuration on the climate and sensitivity of the IPSL-CM5A coupled model. *Clim. Dyn.* **40,** 2167–2192 (2013).
16. Volodin, E. M., Dianskii, N. A. & Gusev, A. V. Simulating present-day climate with the INMCM4.0 coupled model of the atmospheric and oceanic general circulations. *Izv. Atmospheric Ocean. Phys.* **46,** 414–431 (2010).
17. Rotstayn, L. D. *et al.* Aerosol- and greenhouse gas-induced changes in summer rainfall and circulation in the Australasian region: a study using single-forcing climate simulations. *Atmospheric Chem. Phys.* **12,** 6377–6404 (2012).
18. Bentsen, M. *et al.* The Norwegian Earth System Model, NorESM1-M – Part 1: Description and basic evaluation of the physical climate. *Geosci. Model Dev.* **6,** 687–720 (2013).
19. Delworth, T. L. *et al.* GFDL's CM2 Global Coupled Climate Models. Part I: Formulation and Simulation Characteristics. *J. Clim.* **19,** 643–674 (2006).
20. Schmidt, G. A. *et al.* Present-Day Atmospheric Simulations Using GISS ModelE: Comparison to In Situ, Satellite, and Reanalysis Data. *J. Clim.* **19,** 153–192 (2006).

21. Collins, W. J. *et al.* Development and evaluation of an Earth-System model – HadGEM2. *Geosci. Model Dev.* **4,** 1051–1075 (2011).

22. Qiao, F. *et al.* Development and evaluation of an Earth System Model with surface gravity waves: Earth System Model With Wave. *J. Geophys. Res. Oceans* **118,** 4514–4524 (2013).

23. Ji, D. *et al.* Description and basic evaluation of Beijing Normal University Earth System Model (BNU-ESM) version 1. *Geosci. Model Dev.* **7,** 2039–2064 (2014).

24. Arora, V. K. *et al.* Carbon emission limits required to satisfy future representative concentration pathways of greenhouse gases: ALLOWABLE FUTURE CARBON EMISSIONS. *Geophys. Res. Lett.* **38,** L05805 (2011).

25. Li, L. *et al.* The flexible global ocean-atmosphere-land system model, Grid-point Version 2: FGOALS-g2. *Adv. Atmospheric Sci.* 30, 543–560 (2013).
